# CircNF1 modulates the progression and immune evasion of esophageal squamous cell carcinoma through dual regulation of PD-L1

**DOI:** 10.1186/s11658-025-00712-y

**Published:** 2025-03-29

**Authors:** Chang Wang, Chenxi Ju, Dan Du, Peiyu Zhu, Jie Yin, Jinlin Jia, Xue Wang, Xinyu Xu, Li Zhao, Junhu Wan, Ting Sun, Lijun Yang, Hongle Li, Fucheng He, Mingxia Zhou, Jing He

**Affiliations:** 1 Department of Medical Laboratory, The First Affiliated Hospital of Zhengzhou University, Zhengzhou, 450052 Henan China; 2 Key Laboratory of Carcinogenesis and Translational Research, Center of Gastrointestinal Cancer, Peking University Cancer Hospital and Institute, Beijing, 100142 China; 3 Department of Oncology, The First Affiliated Hospital of Zhengzhou University, Zhengzhou, 450052 Henan China; 4 Department of Research and Development, The First Affiliated Hospital of Zhengzhou University, Zhengzhou, 450052 Henan China; 5 Department of Molecular Pathology, The Affiliated Cancer Hospital of Zhengzhou University, Zhengzhou, 450008 China; 6 Department of Gastroenterology, The First Affiliated Hospital of Zhengzhou University, Zhengzhou, 450052 Henan China; 7 Department of Breast Surgery, The First Affiliated Hospital of Zhengzhou University, Zhengzhou, 450052 Henan China

**Keywords:** Esophageal squamous cell carcinoma (ESCC), circRNAs, STAT3, ANXA1, PD-L1

## Abstract

**Background:**

Tumor immune escape is a pivotal gateway for esophageal squamous cell carcinoma (ESCC) development. Immune checkpoint-blocking therapies, represented by programmed cell death receptor-1/ligand 1 (PD-1/PD-L1) inhibitors, have achieved remarkable breakthroughs in ESCC treatment. However, not all patients with ESCC receive satisfactory clinical benefit. Therefore, identifying novel biomarkers for predicting the efficacy of immunotherapy in ESCC is of great importance.

**Methods:**

CircNF1 was screened from the circRNAs microarray, and its expression was measured by droplet digital polymerase chain reaction (ddPCR) and quantitative reverse transcriptase polymerase chain reaction (qRT-PCR) assays in ESCC tissues and serum. Functional experiments were conducted to demonstrate the role of circNF1 in ESCC proliferation, metastasis, and tumor evasion. High-throughput RNA sequencing, chromatin immunoprecipitation (ChIP), co-immunoprecipitation (co-IP), and chromatin isolation by RNA purification-mass spectrometry (ChIRP-MS) were performed to clarify the underlying mechanisms of circNF1-mediated tumor progression.

**Results:**

The upregulation of circNF1 was closely associated with the response of anti-PD-L1 immunotherapy. Functionally, circNF1 promoted ESCC cell malignant phenotypes and regulated CD8^+^ T-cell-mediated antitumor immunity. Mechanistically, circNF1 drove the IL-6-induced oncogenic activation of the JAK–STAT3 pathway, which stimulated p-STAT3 binding of the promoter regions of PD-L1. Furthermore, circNF1 physically interacted with annexin A1 (ANXA1), blocking the ANXA1 deubiquitination induced by ubiquitin-specific protease 7 (USP7), resulting in increased interaction between USP7 and PD-L1 and augmented PD-L1 stability.

**Conclusions:**

Our findings provide novel insights into the specific regulatory mechanism of PD-L1 in ESCC cells, which offer a new strategy for synergizing with anti-PD-L1 therapy.

**Supplementary Information:**

The online version contains supplementary material available at 10.1186/s11658-025-00712-y.

## Introduction

Esophageal cancer ranks sixth in terms of cancer-related mortality and tenth among the most common cancers worldwide, with more than 540,000 deaths and 600,000 new cases estimated annually [1]. Esophageal squamous cell carcinoma (ESCC), characterized by a high risk of recurrence or metastasis and dismal prognosis, constitutes more than 80% of all cases of esophageal cancers [2]. Currently, surgery, chemotherapy, and radiotherapy serve as the mainstay treatment options for ESCC. With the growing understanding of the tumor microenvironment, immunotherapy has renovated the novel treatment strategy for ESCC. A phase 1b trial demonstrated that neoadjuvant PD-L1 blockade with adebrelimab significantly prolonged survival outcomes of patients with advanced ESCC [3]. Another phase 3 study proved that anti-PD-L1 antibody sugemalimab plus chemotherapy extended progression-free survival (PFS) and overall survival (OS) in treatment-naïve late-stage patients with ESCC, indicating the curative efficacy of immune checkpoint blockade therapies [4]. Unfortunately, not all patients with ESCC exhibited good response to anti-PD-L1 therapy. Thus, unraveling the molecular mechanism underlying PD-L1 regulation and identification of predictive biomarkers for immunotherapy efficacy are urgently needed in clinical practice.

Circular RNAs (circRNAs) are a novel class of endogenous noncoding alternatively spliced transcripts, harboring covalently closed circular structures, which have emerged as promising regulators during carcinogenesis [5]. CircRNAs have been demonstrated to act as critical regulators of gene expression in oncogenesis by participating in a variety of molecular functions. These include acting as microRNA (miRNA) sponges, interacting with RNA-binding proteins (RBPs), modulating alternative splicing, regulating transcription, enhancing protein functionality, and even serving as templates for the translation of unique peptides. Their ability to interact with specific miRNAs or RBPs creates downstream effects that influence various hallmarks of cancer, including the activation of proliferative signaling pathways, tumor metastasis, neoangiogenesis, and immune system evasion [6]. Many circRNAs were demonstrated to be involved in the modulation of the programmed cell death receptor-1/ligand 1 (PD-1/PD-L1) pathway in tumor microenvironment. For instance, circBART2.2 was found to promote PD-L1 transcription through binding retinoic acid-inducible gene I (RIG-I), activating transcription factors interferon regulatory factor 3 (IRF3) and nuclear factor-kappa B (NF-κB), eventually inducing immune escape of nasopharyngeal carcinoma [7]. Another study revealed that circIGF2BP3 functioned as the molecular sponge of miR-328-3p and miR-3173-5p to upregulate plakophilin 3 (PKP3) expression, thereby increasing ovarian tumor (OTU) family deubiquitinase ubiquitin aldehyde binding 1 (OTUB1) level and facilitating PD-L1 deubiquitination [8]. In addition, exosomal circRNAs regulate immune pathways, including the PD-1/PD-L1 axis, either by serving as miRNA sponges or by directly influencing the expression and stability of immune checkpoint molecules [9]. However, no prior study has detailed the potential role of circRNAs in the context of PD-L1 regulation in ESCC microenvironment. Identification of circRNAs targeting the PD-1/PD-L1 axis will benefit the tailoring of precise and effective immune strategies in ESCC treatment.

In this study, we uncovered that circNF1, a circRNA generated from the circularization of the neurofibromatosis type 1 (*NF1*) gene, was markedly elevated in ESCC tissues and serum. A series of functional assays indicated that circNF1 could boost ESCC cell proliferation and metastasis and influence antitumor immunity by inducing CD8^+^ T cell dysfunction. Importantly, circNF1 exerted dual regulatory effects on PD-L1 level from transcriptional and posttranslational modification layers. Therefore, our results provide novel insights into the dysregulation of PD-L1 in the ESCC microenvironment and demonstrate circNF1 as a promising immune-response biomarker for ESCC and a therapeutic target for synergizing the efficacy of PD-L1 blockade.

## Materials and methods

### Patient samples

A total of 108 pairs of ESCC tissues and normal adjacent esophagus tissues were harvested from the First Affiliated Hospital of Zhengzhou University and included in cohort 1. The clinical specimens were frozen in liquid nitrogen immediately after collection. A total of 114 patients with ESCC and 101 healthy volunteers’ serum samples were collected from the First Affiliated Hospital of Zhengzhou University and included in cohort 2. Cohort 3 included 60 patients with advanced ESCC, who received neoadjuvant anti-PD-L1 immunotherapy. All clinical specimens were newly diagnosed patients with ESCC who had not received radiotherapy or chemotherapy before surgery. Written informed consent was obtained from each patient in the three cohorts.

### Cell culture

Human ESCC cell lines KYSE150 (cat. no. TCHu236), TE1 (cat. no. TCHu89), and HEK-293 T (cat. no. GNHu17) were purchased from Cell Bank of Chinese Academy of Sciences (Shanghai, China). KYSE70, KYSE450, and esophageal epithelial cell line (HEEC) were obtained from American Type Culture Collection (ATCC, USA). All cells were cultured in Dulbecco’s modified Eagle’s medium or RPMI 1640 medium (NEST Biotechnology, China) supplemented with 10% fetal bovine serum (FBS, BaiDi Biotechnology Co., Ltd.) in a 37 °C incubator containing 5% CO_2_.

### Plasmids and reagents

The plasmids used in this study were synthesized from GENEWIZ (Suzhou, China). The full-length sequence and truncation of ANXA1 (N-terminal and four repeat domains in the C-terminal) were cloned into the pLVX-Myc vector, and the full-length sequence and truncation of USP7 (TRAF, CAT, and TUD) were cloned into the pCDH-CMV-MCS-EF1-Flag vector and confirmed by DNA sequencing. Cycloheximide (CHX) and MG132 were purchased from Selleck (Houston, TX, USA). Stattic was purchased from TargetMol (Boston, MA, USA) and Tocilizumab was purchased from MedChemExpress (Princeton, NJ, USA).

### Transient transfection and lentiviral infection

For siRNA transfection, all siRNA oligonucleotides used in this study were purchased from RiboBio (Guangzhou, China). The sequences of the siRNAs are enumerated in Supplementary Table S1. Transfections were performed using Lipofectamine 3000 reagent (Invitrogen, Carlsbad, CA, USA) in accordance with the manufacturer’s protocol. The recombinant lentivirus for circNF1 (Lv-circNF1) and the nontargeting control lentivirus (Lv-NC) expressing a scrambled RNA were synthesized by GenePharma (Shanghai, China). Cells were incubated and transfected with lentivirus and polybrene (GeneChem Corporation). A total of 2 days after transfection, puromycin (5 μg/mL) was co-cultured with cells to select puromycin-resistant cells and establish stable cell lines with circNF1 overexpression.

### Droplet digital polymerase chain reaction (ddPCR) and quantitative real-time PCR (qRT-PCR)

Total RNA was isolated from tissues and cells using TRIzol reagent (Takara, Japan). Complementary DNA (cDNA) was synthesized with First Strand cDNA Synthesis SuperMix (Novoprotein, Shanghai, China). Droplet digital PCR was performed following the manufacturer’s instructions using One-Step RT-dPCR Master Mix and Enzyme Mix on DQ24 automated ddPCR system (Sniper, Suzhou). The optimal reaction program was established as follows: reverse transcription at 50 °C for 5 min and initial denaturation at 95 °C for 3 min. This was followed by 40 cycles of denaturation at 95 °C for 30 s, and annealing and extension at 60 °C for 30 s. Last, fluorescence signal acquisition was performed at 60 °C for 60 s. The probe sequence of circNF1 was FAM-CTGAGCACAACAAGGAATGTCTAATC-BHQ1. The copies of circNF1 were calculated using Poisson distribution analysis. For qRT-PCR, the obtained cDNA was quantified using Hieff qPCR SYBR Green Master Mix (Yeasen, Shanghai, China) with a QuantStudio 5 Real-Time PCR System (Applied Biosystems, Foster City, CA, USA). The optimal reaction program was determined as 95 °C for 5 min for activation of heat-initiating enzymes, followed by 40 cycles of denaturation at 95 °C for 10 s, and annealing and extension at temperature of 60 °C for 30 s. The relative gene expression was calculated by the 2^−ΔΔCT^ method and normalized by glyceraldehyde-3-phosphate dehydrogenase (*GAPDH*). Primer sequences are listed in Supplementary Table S2. The Cytokines and Chemokines PCR Array was utilized to detect differential expression of 90 cytokines after knockdown of circNF1 (Wcgene Biotech, Shanghai, China), according to the manufacturer’s protocol.

### CircRNA microarray assays

Serum total RNA was extracted from patients with ESCC using the SteadyPure Blood, Serum, and Plasma Small RNA Extraction Kit (Accurate Biology, China). The sample preparation and microarray hybridization were performed on the basis of the Arraystar’s standard protocol. Briefly, serum total RNA was digested with RNase R to remove linear RNA and enrich for circRNA. The enriched circRNA was then amplified and reverse transcribed into fluorescent cDNA using the random primer method. Labelled cRNAs were hybridised nto the Arraystar Human circRNA Array (Aksomics, China) and the arrays were scanned by the Agilent G2505C scanner. Quantile normalization and subsequent data analysis were conducted using R software.

### Protein isolation and western blotting

Cells were lysed in RIPA lysis buffer containing phenylmethyl sulfonyl fluoride (PMSF, Solarbio, Beijing, China) and protease inhibitor (APExBIO, Houston, TX, USA). The concentration of protein was detected by using the BCA Protein Assay Kit (EpiZyme Biotech, Shanghai, China). Protein samples were denatured by boiling with 5× sodium dodecyl sulfate (SDS) loading buffer (Affinibody LifeScience, Wuhan, China), separated on SDS-polyacrylamide gel electrophoresis (PAGE) gel, and passed on polyvinylidene difluoride (PVDF) membranes. The membranes were blocked with 5% nonfat milk, followed by incubation with specified primary antibodies. The protein bands were visualized using enhanced chemiluminescence (ECL) reagent (NCM Biotech, Suzhou, China). Antibodies utilized in this study are specified in Supplementary Table S3.

### Subcellular fractionation

Subcellular fractionation was performed using NE-PER™ Nuclear and Cytoplasmic Extraction Reagents (Thermofisher Scientific, Waltham, MA, USA) according to instructions provided by the manufacturer. RNA from the nuclear and cytoplasmic extracts were extracted using TRIzol reagent. The distribution ratio of circNF1 in the nucleus and cytoplasm was determined by qPCR. U6 was used as the internal standard for nuclear RNA. GAPDH served as a cytosolic marker.

### RNase R treatment and actinomycin D treatment

The circular structure of circNF1 was confirmed by RNase R assay and actinomycin D assay. For RNase R assay, total RNA was divided into two aliquots of 2 μg each and incubated at 37 ℃ for 15 min with or without RNase R (3 U/μg, Geneseed, Guangzhou, China), which was then used for qRT-PCR. For actinomycin D assay, 3 μM actinomycin D (MedChemExpress, Princeton, NJ, USA) was added to KYSE150 and TE1 for 0, 4, 8, 12, and 24 h sequentially, followed by the analysis of the expression level of circNF1 and NF1 mRNA using qRT-PCR.

### RNA FISH assays and immunofluorescence (IF) staining

The Cy3-labeled RNA probes specific for circNF1 were designed by RiboBio (Guangzhou, China) to determine its subcellular localization information. Fluorescence in situ hybridization (FISH) assays were performed with the Fluorescent In Situ Hybridization Kit (RiboBio, Guangzhou, China) following the manufacturer’s protocol. Briefly, cells in the logarithmic growth phases were permeabilized with 0.5% Triton X-100 and blocked with prehybridization buffer at 37 ℃ for 30 min. Hybridization with Cy3-labeled probes was performed overnight in a humidified chamber at 37 °C in the dark. For IF staining, cells were blocked with 5% normal goat serum and then sequentially stained with ANXA1 primary antibody and Alexa-Fluor-488 goat antirabbit immunoglobulin (Ig)G (YEASEN, 33106ES60, 1:100) secondary antibody. After extensive washing steps, cells were stained with 4′,6-diamidino-2-phenylindole (DAPI) for another 10 min. The confocal microscopy imaging and colocalization analysis were conducted using a confocal laser scanning microscope (ZEISS, Aalen, Germany).

### Cell proliferation assays

Cell proliferation was monitored with cell counting kit-8 (CCK-8), 5-ethynyl-2′-deoxyuridine (EdU) assays, and colony-formation assays. For the CCK-8 assay, cells were plated in 96-well plates at a concentration of 2 × 10^3^ cells/well. Then, 10 μL of CCK-8 (Biosharp, Shanghai, China) solution was added to each well at the set time. The absorbance was measured at 450 nm by a microplate reader. The Cell-Light EdU Apollo 488 In Vitro Imaging Kit was purchased from RiboBio (Guangzhou, China). Cells were seeded in 96-well plates at a concentration of 1 × 10^4^ cells/well, and the staining was performed in accordance with the instruction manual. Generally, when the confluence reached 80%, cells were incubated with 50 μM EdU, followed by washing and fixing with 4% formaldehyde for 30 min. Next, the cells labeled with EdU were dyed in Apollo reaction solution, and the nuclei were colored with Hoechst. For colony formation, 1000 cells were seeded in six-well plates and further incubated for 14 days. Finally, the cells were fixed with 4% formaldehyde and stained with 0.1% crystal violet.

### TdT-mediated dUTP Nick-End labeling staining (TUNEL) assays

Cellular apoptosis was assayed using riboAPO One-Step TUNEL Apoptosis Kit (RiboBio, Guangzhou, China) per the manufacturer’s protocol. After fixing with 4% paraformaldehyde for 15 min and increasing membrane permeability with 0.5% Triton X-100 for 2 min, the cells were incubated with TUNEL solution for 30 min. DAPI staining was done using a 1/100 dilution for 30 min at room temperature. Stained cells were imaged using a fluorescent microscope.

### Transwell and wound-healing assays

A total of 4 × 10^4^ cells in 200 μL medium supplemented without FBS were added to the upper chambers. A total of 1 μg/mL Mitomycin C (Sigma, Missouri, MO, USA) was used in transwell assays to offset the growth of ESCC cells. Transwell chambers (Corning, New York, NY, USA) coated with Matrigel were used for the invasion assay. After 48 h of incubation, nonmigratory cells on the upper chamber were gently swiped with cotton swabs, while those on the bottom surface were stained with 0.1% crystal violet and counted using an inverted microscope. For the wound-healing assay, cells were harvested and seeded in 12-well plates and allowed to reach a confluence of approximately 95%. Then, a 20 µL micropipette head was utilized to make a straight scratch. At 0 and 24 h postscratching, the cell wound was photographed with the inverted microscope to calculate the migration area.

### RNA sequencing (RNA-seq)

Total RNA was extracted from TE1 cells transfected with si-NC or si-circNF1, and 1 μg total RNA was used for standard RNA sequencing library preparation. Oligo(dT) beads were used to isolate the poly(A) mRNA. After fragmenting the mRNA, first- and second-strand cDNA were created using random primers. The purified double-stranded cDNA was supplemented with a DA tail, followed by the addition of adapters at both ends via TA ligation. DNA clean beads were then used to select the size of the adapter ligation DNA. Each sample was amplified by PCR with P5 and P7 primers, and the PCR products were verified. Libraries were sequenced by the Illumina HiSeq instrument using a 2× 150 paired-end (PE) configuration.

### Immunohistochemistry (IHC)

IHC was performed using paraffin-embedded tissue sections as described previously [10]. Briefly, sections were deparaffinized and rehydrated and then heated in antigen retrieval buffer at pH 6.0 for 20 min. After blocking, antibodies against ANXA1, PD-L1, antigen kiel 67 (Ki67), cluster of differentiation 8 (CD8), cleaved Caspase-3, tumor necrosis factor alpha (TNF-⍺), granzyme B (GZMB), interferon gamma (IFN-γ), and p-STAT3 were incubated with the sections overnight at 4 °C. After incubating with secondary antibodies for 1 h at 37 °C, sections were finally incubated in 3,3′-diaminobenzidine for 5 min to visualize IHC signal and counterstaining with hematoxylin for 1 min.

### Chromatin immunoprecipitation (ChIP) and RNA-binding protein immunoprecipitation (RIP)

ChIP assay was conducted as previously described [11]. ESCC cells were crosslinked with 1% formaldehyde for 10 min at room temperature and then stopped with 0.125 M glycine. The cell lysate was sonicated on ice to isolate the nucleic fraction. Sonicated chromatin samples were distributed into aliquots. Samples with 2 μg of anti-p-STAT3 antibody were incubated at 4 °C with overnight rotational incubation. Each immunoprecipitation reaction was pulled down by magnetic protein A/G beads (MedChemExpress, Princeton, NJ, USA). Immunoprecipitated DNA was purified and measured by agarose gel electrophoresis and qRT-PCR. RNA immunoprecipitation assays were performed using Magna RIP™ RNA-Binding Protein Immunoprecipitation Kit (Millipore Sigma, Burlington, MA, USA), following the manufacturer’s protocol. Briefly, cells transfected with myelocytomatosis oncogene (Myc)-tagged full-length ANXA1 and its truncated somatic plasmids were collected and lysed with RIP lysis buffer containing protease inhibitors and RNase inhibitors. Cell lysates were incubated with anti-Myc at 4 °C overnight, followed by magnetic beads. The antibody–protein–RNA complexes were captured by magnetic beads, and RNA was extracted using the TRIzol reagent after washing and elution.

### Chromatin isolation by RNA purification-mass spectrometry (ChIRP-MS)

Cells were harvested by trypsin digestion and crosslinked with 3% glutaraldehyde for 30 min at room temperature. Crosslinked cells were then resuspended in the lysis buffer containing protease inhibitors and disrupted by sonication. The supernatant was collected after centrifugation, and 100 pmol biotinylated probes specific to circNF1 were incubated with streptavidin-coated magnetic beads for 30 min. After collecting and washing, the magnetic beads were resuspended in an elution buffer containing Benzonase (20 U) to react at 37 °C for 1 h. The supernatants were de-crosslinked at 95 °C for 30 min, and the proteins obtained were subjected to an LC–MS/MS analysis.

### Co-immunoprecipitation assay (co-IP) and ubiquitination assays

Cell lysates were preconjugated with the indicated primary antibodies or isotype control IgG for 6 h. The protein–antibody mixtures were then co-incubated with protein A/G magnetic beads overnight at 4 °C. The magnetic beads were washed using nonidet P-40 (NP-40) buffer and then boiled for 10 min in 2× SDS-PAGE loading buffer to elute protein complexes. Immunoprecipitates were analyzed using western blotting. For ubiquitination assays, cells were harvested after 8 h of incubation with the 10 μM proteasome inhibitor MG132. The cells were then lysed with NP-40 lysis buffer and immunoprecipitated as described above. The co-IP products were used for western blotting using anti-Ub primary antibodies.

### Peripheral blood mononuclear cell (PBMC) isolation and co-culture assays

PBMCs were isolated by Ficoll gradient using separation tubes (NEST Biotechnology) from the whole blood of healthy donors. A total of 1 × 10^7^ PBMCs were stimulated for 24 h in the presence of 2 μg/mL CD3 antibody, 1 μg/mL CD28 antibody (BioLegend, San Diego, CA, USA), and 100 IU/mL recombinant human IL-2 (Solarbio, Beijing, China). KYSE150 and TE1 cells with stable overexpressing circNF1 were seeded in 24-well plates. After overnight culture, activated T cells were added at a ratio of 5:1 and co-cultured for 18 h. Surviving tumor cells were collected for fixation and stained with 0.1% crystal violet. PBMCs were collected and inoculated into polylysine-coated 24-well plates for wall attachment for EdU or TUNEL staining.

### Enzyme-linked immunosorbent assay (ELISA)

The levels of TNF-α, granzyme B, and IFN-γ in cell culture supernatants were measured by commercial ELISA kits (R&D Systems, USA and Beyotime, China) according to the manufacturer’s protocols, and the concentration of the corresponding cytokines was calculated by testing the absorbance at 450 nm.

### Animal experiments

All female NOD-SCID-gamma (NSG)-MHC I/II DKO mice were purchased from GemPharmatech (Jiangsu, China). PBMCs from healthy donor sources were diluted at a ratio of 1:2 with phosphate-buffered saline (PBS) and then injected into the tail vein of NSG mice (1 × 10^5^ per mouse). After 15 days, TE1, specially treated according to experimental requirements, was injected subcutaneously into NSG mice that established a simulated immune system (5 × 10^6^ per mouse). When the tumor volume reached approximately 50 mm^3^, genOFFTM in vivo siRNA (RiboBio, Guangzhou, China) (5 nmol/mouse) or PD-L1 monoclonal antibody (mAb) (BioXcell, New Hampshire, USA) (10 mg/kg) was administered by intratumorally injection every 3 days and six times in total. The volume of the tumor was measured every 6 days and calculated using the following formula: width^2^ × length / 2. At the termination of the experiment, the mice were sacrificed, and the tumors were removed for subsequent experiments.

### Statistical analysis

All data were derived from three independent experiments, analyzed using GraphPad Prism 9 software, and presented as mean ± standard deviation (SD). Differences between the two groups were examined using Student’s *t*-test according to the results of normality and homogeneity tests of variance. Comparisons of clinicopathologic characteristics were subjected to the chi-squared test. For all tests, we considered a *P*-value of less than 0.05 as statistically significant.

## Results

### Identification and characterization of circNF1 in ESCC

**Fig. 1:**
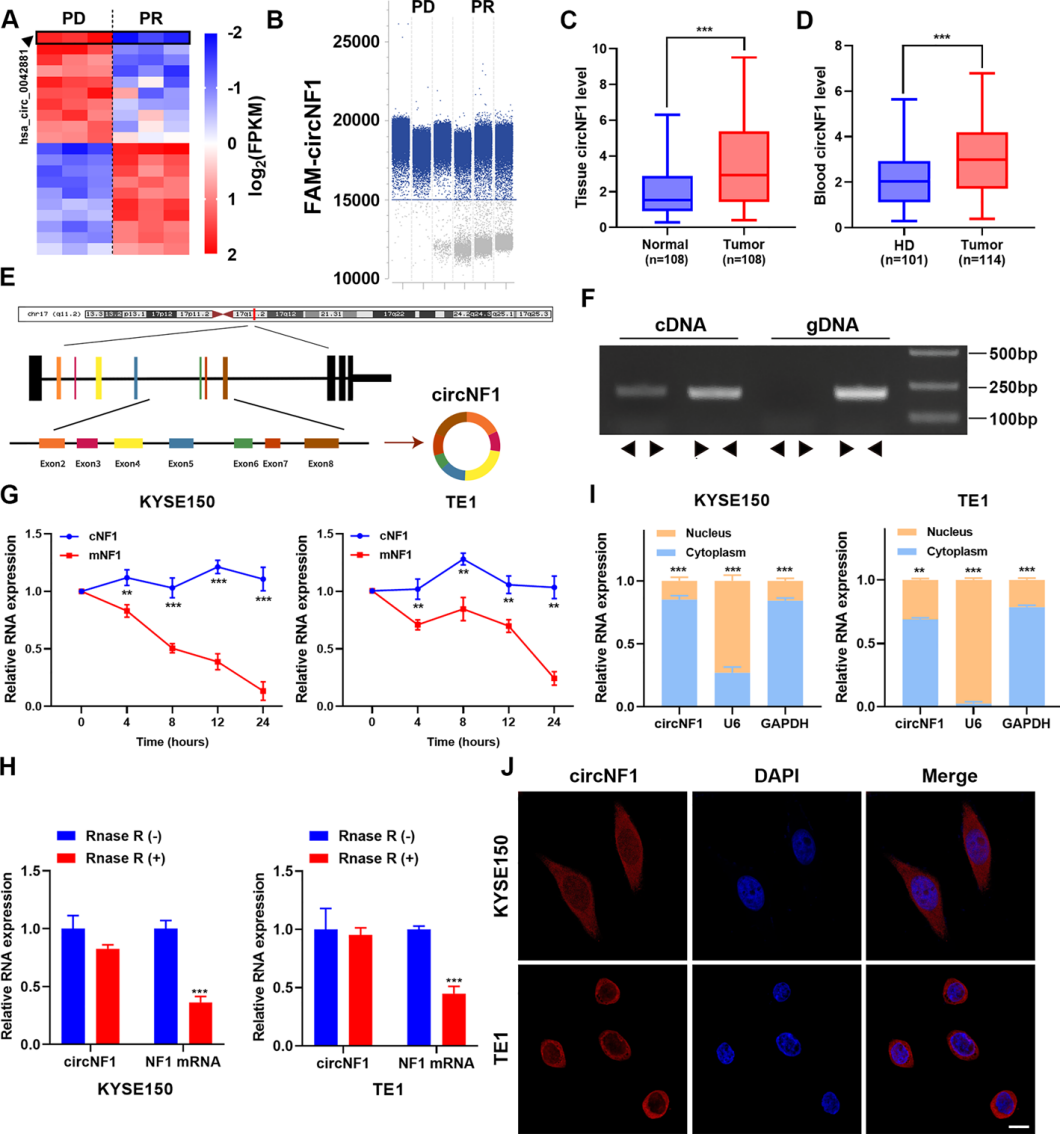
CircNF1 characteristics and expression in ESCC. **A** Heat map of differentially expressed circRNAs between the serum of PD and PR patients by human circular RNA Array (circRNAs top ten upregulated and downregulated). **B** Dot plot representing the positive PCR amplification droplets (blue) and negative droplets without amplification (grey) of circNF1 in patients with different anti-PD-L1 treatment response. **C** Differential abundance of circNF1 between primary ESCC tissues compared with paired normal esophagus tissues (*n* = 108). **D** Expression levels of circNF1 in serum from patients with ESCC (*n* = 114) and healthy donors (*n* = 101). **E** Schematic representation of the genomic locus and structure of circNF1. **F** Divergent and convergent primers were used to amplify circNF1 in cDNA and gDNA in KYSE150 cells. **G** Actinomycin D treatment was performed to assess the stability of circNF1 and NF1 in ESCC cells. **H** The relative expression of circNF1 and NF1 mRNA after Rnase R treatment. **I** qRT-PCR analysis of circNF1 in the nuclear/cytoplasmic fraction of ESCC cells. **J** Representative FISH images of circNF1 (red) in KYSE150 and TE1 cells. Blue, DAPI staining of nuclei. Scale bar: 10 μm. The data are presented as mean ± SD. **P < 0.01, ***P < 0.001

To screen for the potential biomarkers specifically linked to the anti-PD-L1 therapy efficacy, we collected the serum of three patients with ESCC with progressive disease (PD) and three patients with ESCC who achieved partial response (PR) after two cycles of anti-PD-L1 immunotherapy for human circular RNA array. Notably, one circRNA circ_0042881, also named circNF1, drew our attention. CircNF1 exhibited obvious differential expression between patients with PR and PD and was profoundly decreased in patients with a favorable response rate of PD-L1 (Fig. 1A). Droplet digital polymerase chain reaction (ddPCR) was conducted to acquire the absolute quantification of circNF1 in these patients for expression verification (Fig. 1B and Supplementary Fig. S1A). Subsequently, we evaluated circNF1 expression in tumor tissues (cohort 1) and serum (cohort 2) of patients with ESCC via ddPCR and qRT-PCR. Expression profiles indicated that circNF1 displayed elevated expression in ESCC tissues and serum, as measured by ddPCR (Supplementary Fig. S1B and C). Consistently, among the 108 pairs of samples tested, the level of circNF1 in ESCC tissues was robustly higher than that in adjacent normal tissues and was relevant to the lymph node metastasis of patients with ESCC (Fig. 1C and Table S4). We also observed that circNF1 had a higher abundance in the serum of patients with ESCC compared with the healthy donors (Fig. 1D). A receiver operating characteristic curve (ROC) was plotted to analyze the diagnostic value of circNF1 in tumor tissues and the serum of patients with ESCC; the areas under the curve (AUC) were 0.7023 and 0.6680, respectively (Supplementary Fig. S1D). Fluorescence in situ hybridization (FISH) experiments demonstrated circNF1 upregulation in ESCC tissues (Supplementary Fig. S1E). CircNF1 was also highly expressed in ESCC cell lines compared with normal esophagus epithelial cell HEEC (Supplementary Fig. S1F). CircNF1 is located at chr17:29,483,000–29509683, formed by back-splicing of exons 2 to 8 of the neurofibromatosis type 1 (*NF1*) gene (Fig. 1E). Given that circNF1 has a unique ring structure different from the NF1 linear transcript, we designed divergent and convergent primers for circNF1 and then performed agarose gel electrophoresis to detect the amplification of PCR products. The result revealed that the template sequence indispensable for circNF1 amplification with divergent primers was present in cDNA, but not in gDNA (Fig. 1F). Next, KYSE150 and TE1 cells were treated with actinomycin D to assess the degradation of NF1 linear and circular transcripts. qRT-PCR data indicated that the half-life of NF1 circular transcript was longer than that of linear transcripts upon inhibition of the transcription process (Fig. 1G). In addition, circNF1 was more resistant to RNase R than NF1 mRNA owing to the lack of 5′ caps and 3′ polyA tails (Fig. 1H). The above experiments suggested that the structure and properties of circNF1 were consistent with other prevalent circRNAs. Finally, FISH and mRNA fractionation assays disclosed that circNF1 was mainly localized in the cytoplasm in KYSE150 and TE1 cells, although a fraction was present in the nucleus (Fig. 1I-J).

### CircNF1 impacts ESCC cell proliferation, migration, and invasion

**Fig. 2:**
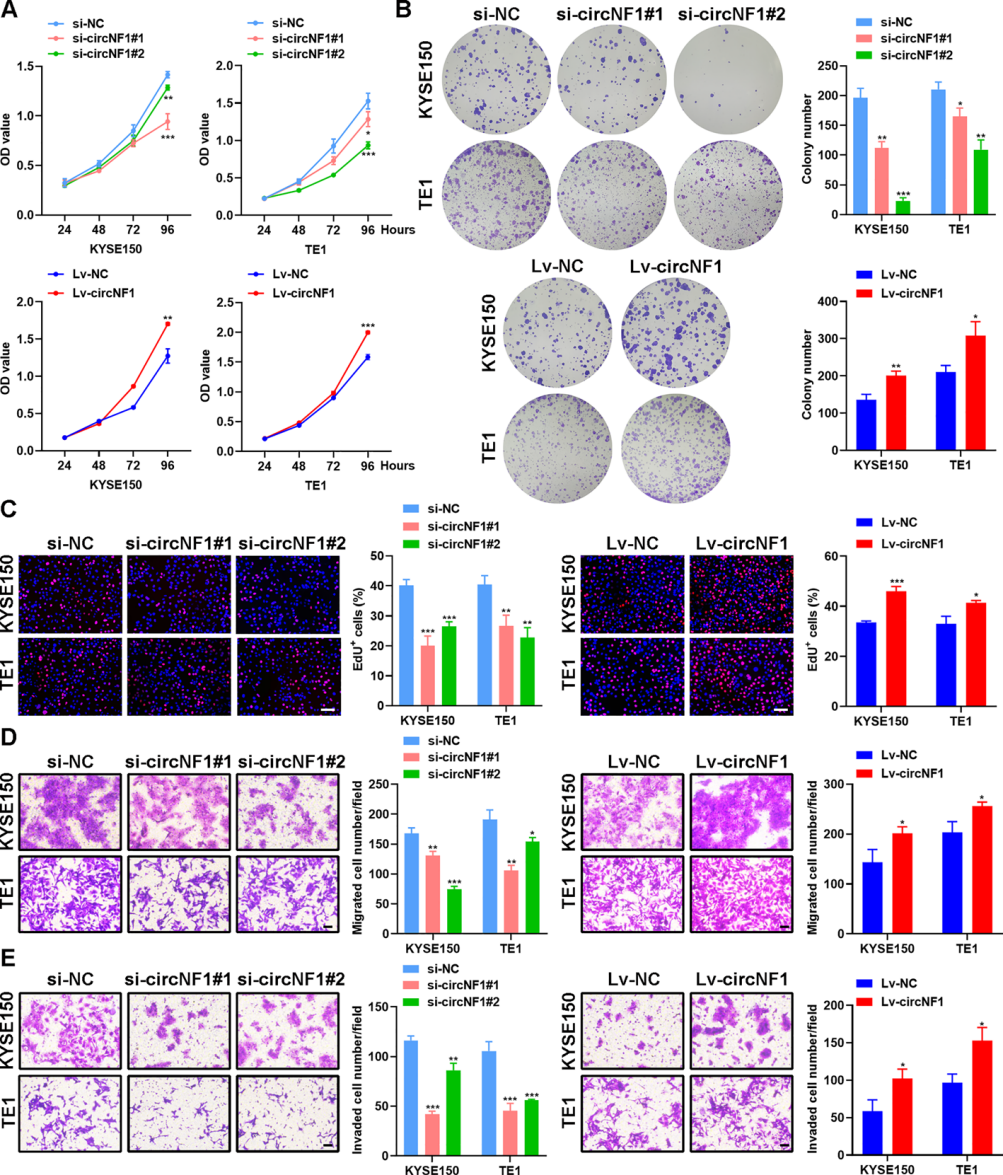
CircNF1 promotes ESCC proliferation, migration and invasion in vitro. **A** CCK-8 assays evaluating the proliferation abilities of ESCC cells after circNF1 knockdown or overexpression. **B** Detection of ESCC cell proliferation after circNF1 knockdown or overexpression by colony formation assays. **C** EdU assays were used to assess the effects of circNF1 knockdown or overexpression on the proliferation abilities of ESCC cells. Scale bar: 100 μm. **D**, **E** Transwell migration (**D**) and invasion **E** assays measuring the migration and invasion abilities in ESCC cells knocking down or overexpressing circNF1. Scale bar: 100 μm. The data are presented as mean ± SD. *P < 0.05, **P < 0.01, ***P < 0.001

With the abnormal expression and diagnostic significance of circNF1 in ESCC, we proceeded to explore whether circNF1 regulates ESCC malignancies in vitro. To artificially reduce circNF1 levels in ESCC cells, we designed three siRNAs specifically targeting circNF1 and then verified the knockdown efficiency by qRT-PCR. We found that si-circNF1#1 and si-circNF1#2 were more effective in diminishing circNF1. Accordingly, these two siRNAs were applied to subsequent experiments (Supplementary Fig. S2A). Next, the full-length sequence of circNF1 was constructed into the GV689 lentiviral vector, followed by puromycin screening to obtain cells stably overexpressing circNF1 (Supplementary Fig. S2B). Notably, neither knockdown nor overexpression of circNF1 altered NF1 mRNA levels. We performed CCK-8, colony-formation, and EdU assays to evaluate the proliferative capacity of ESCC cells. The results indicated that after circNF1 knockdown, cell viability was decreased, the percentage of ESCC cells in the replicative phase was reduced, and clone formation was diminished, all of which indicated the repression of cell proliferation. In contrast, elevated circNF1 expression contributed to ESCC cell proliferation (Fig. 2A-C). Transwell assays and wound-healing experiments demonstrated that the reduction in migration and invasive capacities of KYSE150 and TE1 cells was significantly blunted by siRNA-mediated knockdown of circNF1, with contrasting results obtained in circNF1 stably overexpressing cells (Fig. 2D–E and Supplementary Fig. S2C–D). In conclusion, we observed a positive effect of circNF1 levels on proliferation, migration and invasion of ESCC cells in vitro.

### CircNF1-mediated STAT3 phosphorylation drives PD-L1 transcription and promotes ESCC progression

**Fig. 3:**
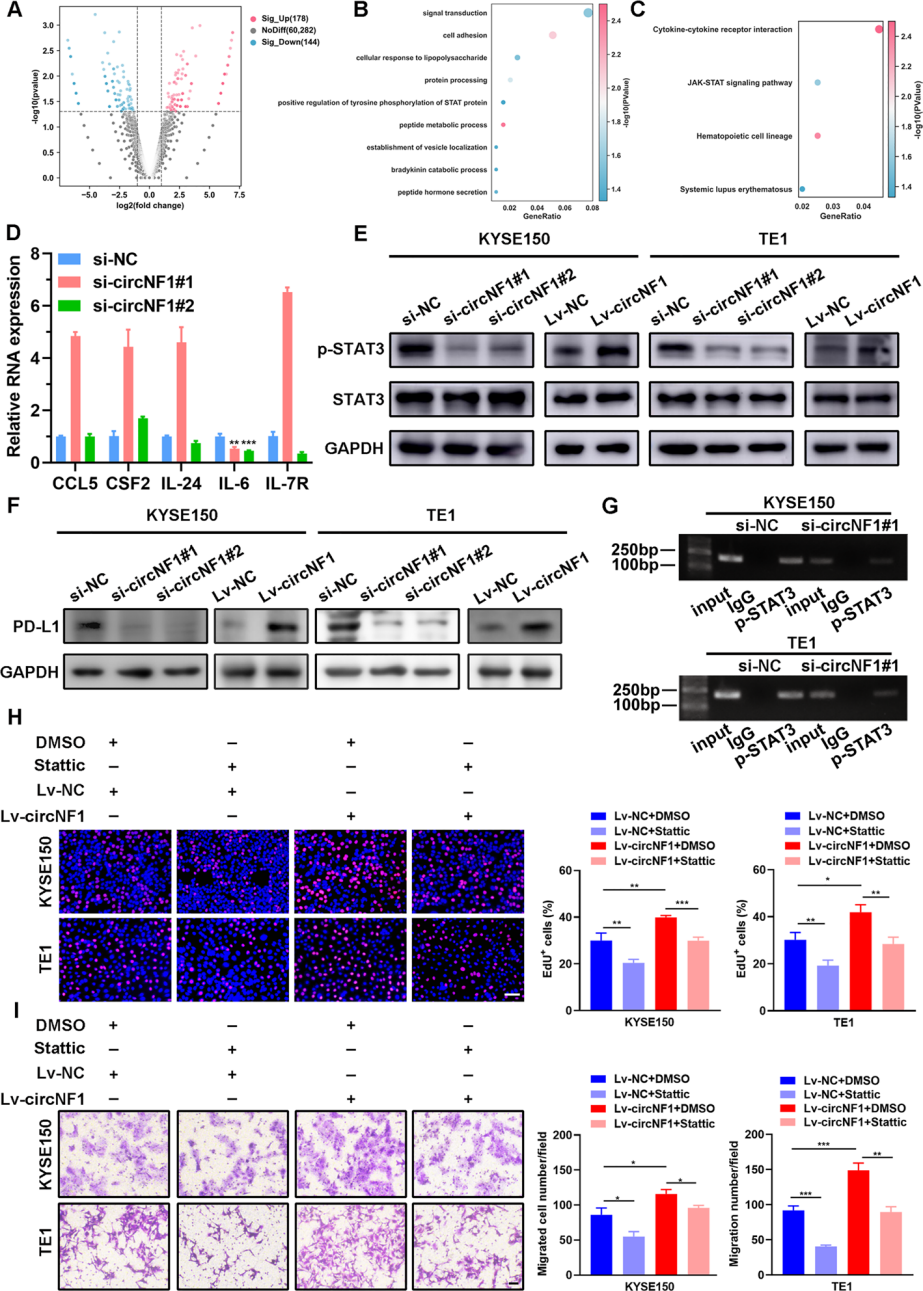
CircNF1 manipulates the JAK–STAT3 pathway to regulate ESCC malignant progression and PD-L1 transcription. **A** Volcano plot showing differentially expressed genes in TE1 cells transfected with si-circNF1 and si-NC. **B**, **C** GO analysis **B** and KEGG pathway analysis (**C**) of circNF1-regulated genes in RNA-seq dataset. **D** qRT-PCR validating the expression of five candidate genes involved in the JAK–STAT3 pathway in circNF1 knockdown cells. **E** Western blotting assay detecting the expression of STAT3 and p-STAT3 in circNF1 knockdown or overexpressing cells, with GAPDH as normalized control. **F** Western blotting assay detecting PD-L1 expression in circNF1 knockdown or overexpressing cells, with GAPDH as normalized control. **G** ChIP assays analyzing whether p-STAT3 is bound to the PD-L1 promoter region in KYSE150 and TE1 cells. **H**, **I** EdU (**H**) and Transwell (**I**) assays evaluating the proliferation and migration ability of ESCC cells treated with DMSO or Stattic in control and circNF1-overexpressed ESCC cells. Scale bar: 100 μm. The data are presented as mean ± SD. *P < 0.05, **P < 0.01, ***P < 0.001

To delineate the potential pathways linked to circNF1, bulk RNA-seq analysis was carried out in circNF1-silenced TE1 cells. Compared with the control cells, 178 genes were upregulated, while 144 genes were downregulated in circNF1 knockdown cells (Fig. 3A). Gene ontology (GO) and gene set enrichment analysis (GSEA) revealed that the differentially expressed genes were associated with various biological processes, including signal transduction, cell adhesion, and T cell activation involved in immune response, which are critical for tumor cell fate (Fig. 3B and Supplementary Fig. S3A). Combined with the results of pathway enrichment analysis, we were interested in one of the signaling pathways, the JAK–STAT3 signaling pathway, as its dysregulation is closely associated with immune activation and cancer progression (Fig. 3C). Similar bioinformatics findings were also verified in our GSEA analysis (Supplementary Fig. S3B). Since cytokines and their receptors are major activators of the JAK–STAT3 signaling pathway, we used the Cytokines and Chemokines PCR Array to screen for differentially expressed cytokines after circNF1 knockdown (Supplementary Fig. S3C). In addition, qRT-PCR assays were utilized for further validation of the cytokines enriched in the JAK–STAT3 pathway (Fig. 3D and Supplementary Fig. S3D). Our comprehensive analysis of the above results revealed that IL-6 was markedly influenced by circNF1 knockdown. As is known, IL-6 functions as the activator of the canonical STAT3 pathway mediating cancer cell proliferation and metastasis as well as the suppressor of tumor immune responses. Western blotting indicated a significant decline in p-STAT3 (Tyr705) level in KYSE150 and TE1 cells after circNF1 knockdown, whereas forced expression of circNF1 produced the opposite effect. However, the total STAT3 protein level remained unaffected by circNF1 (Fig. 3E). To further validate that circNF1 regulates STAT3 phosphorylation through IL-6, we treated ESCC cells with tocilizumab to block the binding of IL-6 and IL-6R. As shown in Supplementary Fig. S3E, tocilizumab treatment resulted in a substantial decrease in the level of p-STAT3, regardless of whether or not circNF1 was overexpressed, and there was no significant difference between the control and circNF1 overexpression groups. Since cirNF1 expression was related to the T cell-mediated immune response, and previous evidence indicated that STAT3 could serve as an upstream regulator of PD-L1, we wondered whether circNF1 could drive PD-L1 expression by phosphorylating STAT3 [12]. As depicted in Fig. 3F, circNF1 knockdown or overexpression resulted in a corresponding decrease or increase in PD-L1 level, respectively. Next, ChIP assay was conducted to investigate the interaction between p-STAT3 and PD-L1 in ESCC cells. Notably, PD-L1 was abundantly enriched by anti-p-STAT3, while ablation of circNF1 reduced the occupation of STAT3 on the promoter region of PD-L1 (Fig. 3G and Supplementary Fig. S3F). To reinforce the reliability of the above findings, we treated ESCC cells with STAT3 phosphorylation inhibitor Stattic (Supplementary Fig. S3G). Treatment with Stattic attenuated the bolstered level of PD-L1 caused by circNF1 overexpression in KYSE150 and TE1 cells (Supplementary Fig. S3H). We assessed whether the STAT3 pathway participated in the circNF1-triggered proliferative and metastatic ability of ESCC cells. EdU and CCK-8 assays demonstrated that the Stattic treatment abolished the proliferation of ESCC cells stably overexpressing circNF1 (Fig. 3H and Supplementary Fig. S3I). Furthermore, wound-healing and transwell assays revealed that the blockade of STAT3 partially attenuated the promotion of ESCC cell migration and invasion by circNF1 (Fig. 3I and Supplementary Fig. S3J–K). Collectively, these data suggested that circNF1 phosphorylated STAT3 via promoting the secretion of IL-6, thereby altering the enrichment level of p-STAT3 on the PD-L1 promoter, eventually driving PD-L1 transcription and accelerating the malignant progression of ESCC cells.

### CircNF1 destabilizes ANXA1 protein by promoting ubiquitin proteasome-mediated ANXA1 degradation

**Fig. 4:**
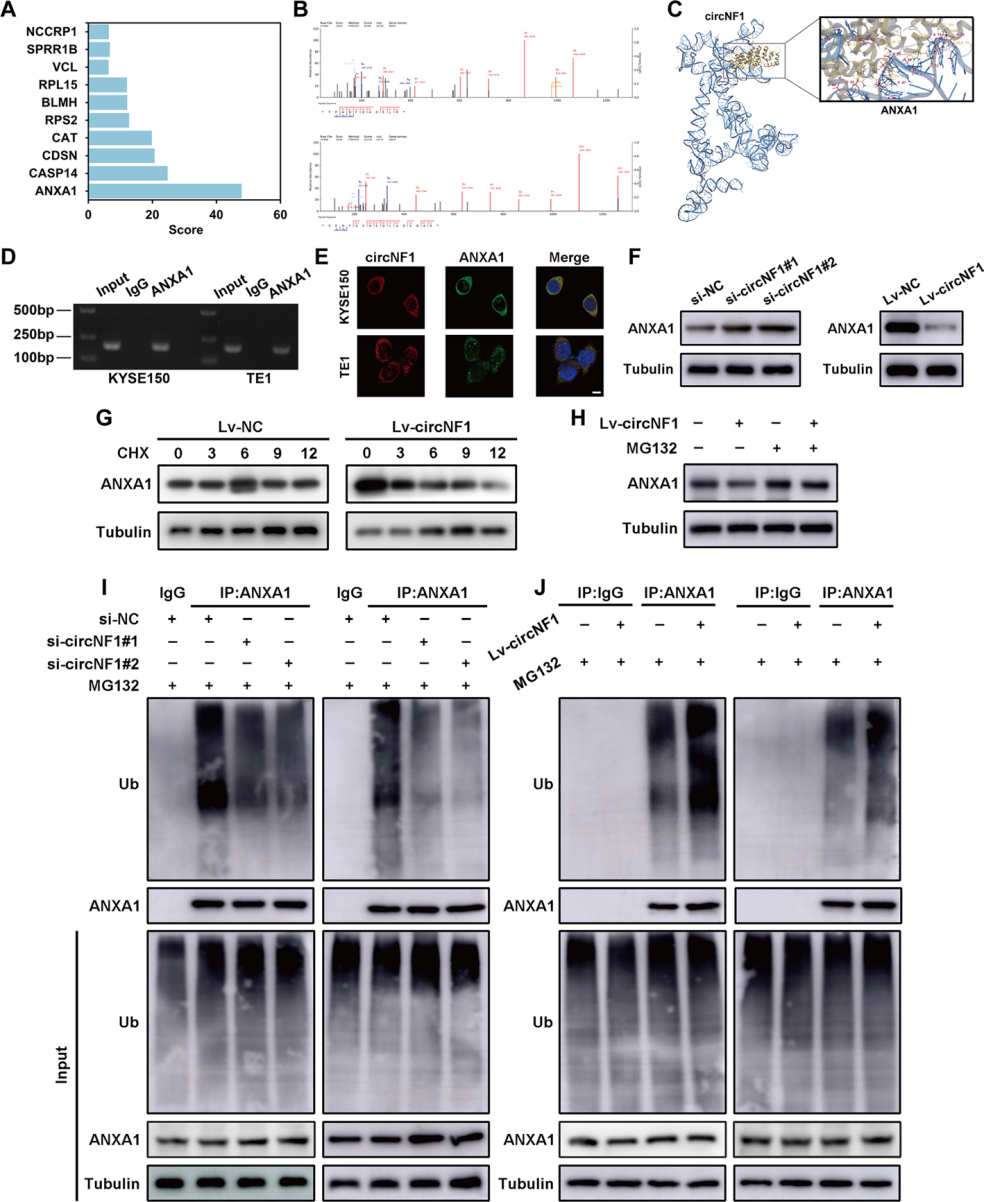
CircNF1 physically binds to ANXA1 and accelerates its degradation via the ubiquitin–proteasome pathway. **A** List and scoring of circNF1-binding proteins. **B** Secondary mass spectrometry of the top two ANXA1 scoring peptides. **C** Docking models of circNF1–ANXA1 complexes. **D** RIP assays with antibody against ANXA1 to pull down circNF1 in ESCC cells. **E** Confocal images of circNF1 (red) together with ANXA1 (green) in TE1 cells. DAPI staining of nuclei. Scale bar: 10 μm. **F** Western blotting detecting ANXA1 protein level in KYSE150 cells after knocking down or overexpressing circNF1. **G** Western blotting showing the effect of overexpressing circNF1 on ANXA1 protein stability in KYSE150 cells treated with 20 µg/mL CHX for the indicated times. *H* Western blotting analyzing ANXA1 protein level after treatment with proteasome inhibitor MG132 in circNF1-overexpressed KYSE150 cells. *I*, *J* Co-IP showing ANXA1 polyubiquitination level after circNF1 knockdown (*I*) and overexpression (*J*) in KYSE150 (left panel) and TE1 (right panel) cells

To discover the interacting partner underlying the oncogenic role of circNF1 in ESCC, we performed ChIRP-MS assays and then captured ten proteins capable of binding circNF1 (Fig. 4A). To ascertain protein scores on the basis of peptide posterior error probabilities, we focused on ANXA1, which had the highest score, for mechanistic studies. In total, seven unique peptides were identified from ANXA1 on the basis of their high specificity (Fig. 4B and Supplementary Fig. S4A). We employed AlphaFold2 to predict the crystal structure of ANXA1 and 3dRNA modeling to predict the tertiary structures of circNF1. Then, molecular docking of ANXA1 and circNF1 was performed using the nucleotide–protein docking module of Schrodinger software (Fig. 4C). In addition, RIP assay further confirmed that ANXA1 was indeed capable of binding circNF1 (Fig. 4D). We also performed FISH-immunofluorescence (FISH-IF) analysis observing that circNF1 colocalized with ANXA1 in the cytoplasm of ESCC cells (Fig. 4E). GEPIA database revealed that ANXA1 expression was downregulated in ESCC, in contrast to circNF1 (Supplementary Fig. S4B). To determine whether the circNF1-ANXA1 complex exerted an influence on ANXA1 expression, we conducted qRT-PCR and western blotting, demonstrating that circNF1 did not affect the mRNA level of ANXA1, whereas it could negatively regulate ANXA1 protein level (Fig. 4F and Supplementary Fig. S4C–D). We used CHX to block protein synthesis in a timecourse experiment and detected that ANXA1 protein in ESCC cells stably overexpressing circNF1 exhibited a shortened half-life, suggesting that circNF1 may affect ANXA1 stability (Fig. 4G and Supplementary Fig. S4E). To further verify the hypothesis, we treated ESCC cells with MG132, a specific inhibitor for proteasome, and then examined ANXA1 expression in the presence or absence of overexpressing circNF1. The results showed that the MG132 treatment abolished the suppression of ANXA1 expression by circNF1 (Fig. 4H and Supplementary Fig. S4F). Specifically, immunoprecipitation assays using anti-ubiquitin antibodies demonstrated that the deletion of circNF1 clearly blocked ANXA1 ubiquitination, whereas ectopically expressed circNF1 elevated ANXA1 ubiquitination in ESCC cells (Fig. 4I-J). Altogether, circNF1 physically interacted with ANXA1 and promoted the ubiquitin–proteasome-mediated degradation of ANXA1. We then assessed the proliferative, migratory, and invasive abilities of KYSE150 and TE1 cells to ascertain whether circNF1 could promote ESCC progression in an ANXA1-dependent manner. The knockdown of circNF1 inhibited ESCC proliferation, migration, and invasion in vitro, which could be partially reversed by ANXA1 downregulation (Supplementary Fig. S5A–D).

### CircNF1 facilitates PD-L1 expression by mediating the physical interaction between USP7 and PD-L1

**Fig. 5:**
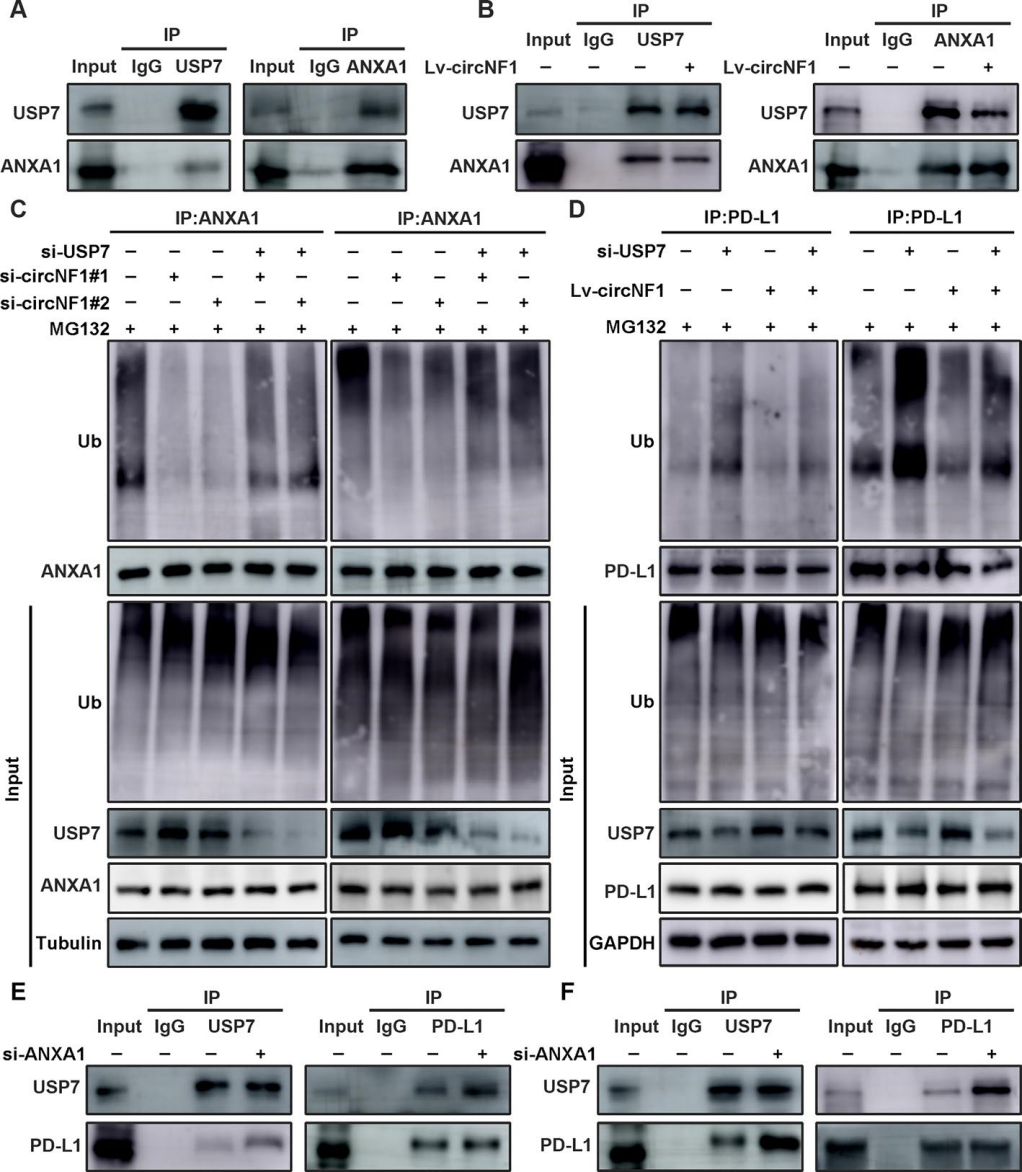
CircNF1 blocks USP7 binding to ANXA1, leading to increased binding between USP7 and PD-L1. **A** Co-IP assays were performed using antibodies against USP7 and ANXA1 in KYSE150 cells. **B** Co-IP analysis of the interaction between USP7 and ANXA1 in circNF1-overexpressed KYSE150 cells and control cells. **C** Detection of ANXA1 polyubiquitination level in KYSE150 (left panel) and TE1 (right panel) cells transfected with si-USP7 or si-circNF1 using immunoprecipitation. **D** Detection of PD-L1 polyubiquitination level in KYSE150 (left panel) and TE1 (right panel) cells transfected with si-USP7 or overexpressed circNF1 using immunoprecipitation. **E**, **F** Co-IP analysis of the interaction between USP7 and PD-L1 in control and ANXA1-deficient KYSE150 (left panel) and TE1 (right panel) cells

Considering that ANXA1 has been reported to be the substrate of deubiquitinating enzyme USP7, we speculated that circNF1 may interfere with the process of ANXA1 deubiquitination by USP7 [13]. With this aim, we first confirmed that circNF1 levels did not affect the USP7 protein expression, as measured by western blotting (Supplementary Fig. S6A). Next, we performed co-IP experiments and proved the specific binding of USP7 and ANXA1 (Fig. 5A and Supplementary Fig. S6B). As expected, the knockdown of USP7 dramatically increased ubiquitinated ANXA1 (Supplementary Fig. S6D). However, endogenous co-precipitation of USP7 and ANXA1 was impaired after ectopic expression of circNF1, indicating that circNF1 damaged the interaction between USP7 and ANXA1 (Fig. 5B and Supplementary Fig. S6C). Immunoprecipitation results showed that circNF1 knockdown markedly reduced the ubiquitination level of ANXA1, whereas in USP7-deleted cells, the attenuation of ANXA1 ubiquitination induced by decreased circNF1 levels was remedied (Fig. 5C). These results demonstrated that circNF1 inhibited USP7-mediated deubiquitination of ANXA1, thereby decreasing ANXA1 stability and suppressing its expression. Several studies have reported that USP7 also acts as a deubiquitinating enzyme for PD-L1 [14]. Similar findings were validated in the ESCC cells using an in vitro ubiquitination assay (Supplementary Fig. S6E). Interestingly, we determined that circNF1 restricted PD-L1 ubiquitination modification levels, while this effect could be prevented after silencing of USP7 (Fig. 5D). Accordingly, we proposed the hypothesis that circNF1 could alter PD-L1 ubiquitination by modulating ANXA1 binding to USP7. Consistent with this, a stronger interaction between PD-L1 and USP7 was observed when ANXA1 levels were reduced or circNF1 levels were increased (Fig. 5E–F and Supplementary Fig. S6F–G).

**Fig. 6:**
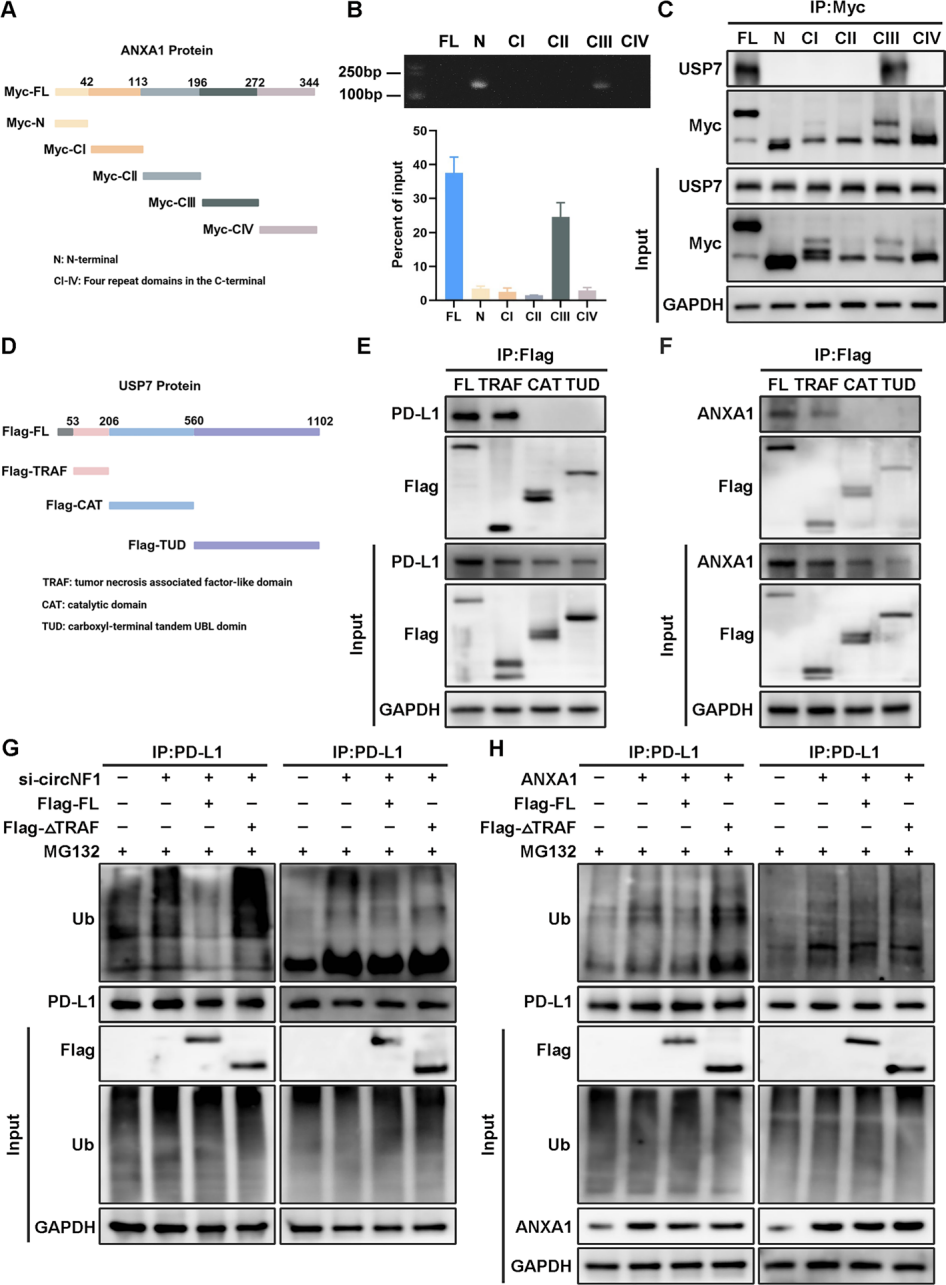
Spatially competitive binding of ANXA1 by circNF1 decreases polyubiquitination modification of PD-L1. **A** Schematic representation of ANXA1 protein structure and its truncation constructs. **B** RIP assays validating the ectopic expression of Myc-tagged ANXA1 (full-length or short truncations) binding to circNF1 were presented using agarose gel electrophoresis (upper panel) or qRT-PCR (bottom panel) after immunoprecipitation in KYSE150 cells. **C** Co-IP revealing that the third repeat domain at the C-terminus of ANXA1 protein interacted with USP7 in KYSE150 cells. **D** Schematic representation of USP7 protein structure and its truncation constructs. *E*, *F* Co-IP showing the detailed region of USP7 bound to ANXA1 (**E**) or PD-L1 (*F*) in KYSE150 cells. **G** Co-IP detecting the antagonism of full-length or TRAF domain-deficient USP7 on upregulated endogenous PD-L1 ubiquitination caused by knocking down circNF1 in KYSE150 (left panel) and TE1 (right panel) cells. **H** Co-IP detecting the antagonism of full-length or TRAF domain-deficient USP7 on upregulated endogenous PD-L1 ubiquitination caused by ectopic expression of ANXA1 in KYSE150 (left panel) and TE1 (right panel) cells

To elucidate the detailed molecular mechanisms underlying the aforementioned phenomenon, we first constructed the Myc-tagged ANXA1 plasmid and its truncated plasmids (Fig. 6A). RIP assay performed in transfected cells revealed that circNF1 could bind to the C-terminal third repeat domain of ANXA1 (Fig. 6B). Next, co-IP experiments using anti-Myc antibodies were conducted to determine the specific interaction sites between ANXA1 and USP7. Strikingly, the C-terminal third repeat domain of ANXA1 was also responsible for the USP7 binding (Fig. 6C). The above findings provided the rationale for the increased ANXA1 ubiquitination caused by circNF1. CircNF1 spatially occupied the same domain of ANXA1 that was bound to USP7, thereby suppressing the interaction of USP7 with ANXA1. Given that both ANXA1 and PD-L1 could serve as the substrates for USP7, we sought to distinguish the domain of USP7 that was bound to ANXA1 or PD-L1, respectively. USP7 consists of three structural domains: the tumor necrosis associated factor-like domain (TRAF), catalytic domain (CAT), and carboxyl-terminal tandem UBL domain (TUD). We constructed functional truncations of these three structural domains carrying a Flag tag (Fig. 6D). Immunoprecipitation assays revealed that PD-L1 interacted with TRAF truncation of USP7 independently of the other two regions (Fig. 6E). Similar observations were made in the binding patterns between ANXA1 and USP7, implying that ANXA1 and PD-L1 are competitively bound to the same domain of USP7 (Fig. 6F). Furthermore, overexpression of the full-length USP7 sequence dramatically ameliorated the elevated PD-L1 ubiquitination mediated by circNF1 knockdown or ANXA1 overexpression, whereas these effects vanished in the group of cells transfected with USP7 sequence lacking TRAF domain (Fig. 6G and H). In conclusion, our research uncovered a novel mechanism for USP7- and ANXA1-maintained PD-L1 stability and highlighted the central role of circNF1 in this process.

### CircNF1 promotes tumor immune escape in ESCC cells

It is well-recognized that tumor cells can evade the immune system surveillance by hyperactivating PD-L1. As the above results indicated that circNF1 stabilized PD-L1 abundance in ESCC cells, we evaluated whether circNF1 could mediate tumor immunosuppression. The co-incubation model was constructed by isolating PBMCs in human whole blood and co-culturing them with KYSE150 and TE1 cells (Supplementary Fig. S7A). In the T cell-mediated killing assay, circNF1 overexpression reduced the sensitivity of ESCC cells to activated T cell-mediated cytolysis (Supplementary Fig. S7B). Next, we compared the immunosuppressive effects of Lv-circNF1 and control cells on PBMCs. As shown in Supplementary Fig. S7C-D, co-culture with circNF1-overexpressing ESCC cells led to attenuated proliferation and increased apoptosis of PBMCs, as evidenced by EdU and TUNEL experiments. We next analyzed whether the levels of the immune effectors in PBMCs were impacted after co-culture with circNF1 aberrantly expressing ESCC cells. Importantly, western blotting and ELISA experiments revealed that circNF1 knockdown induced co-cultured T cells to secrete more TNF-α, IFN-γ, and GZMB, whereas circNF1 overexpression resulted in compromised levels of these cytotoxicity-related molecules (Supplementary Fig. S7E–F). Taken together, these data suggested that circNF1 suppressed antitumor immunity by altering the cytotoxicity of T cell activity.

### Targeting circNF1 synergizes with PD-L1 checkpoint blockade in ESCC

**Fig. 7:**
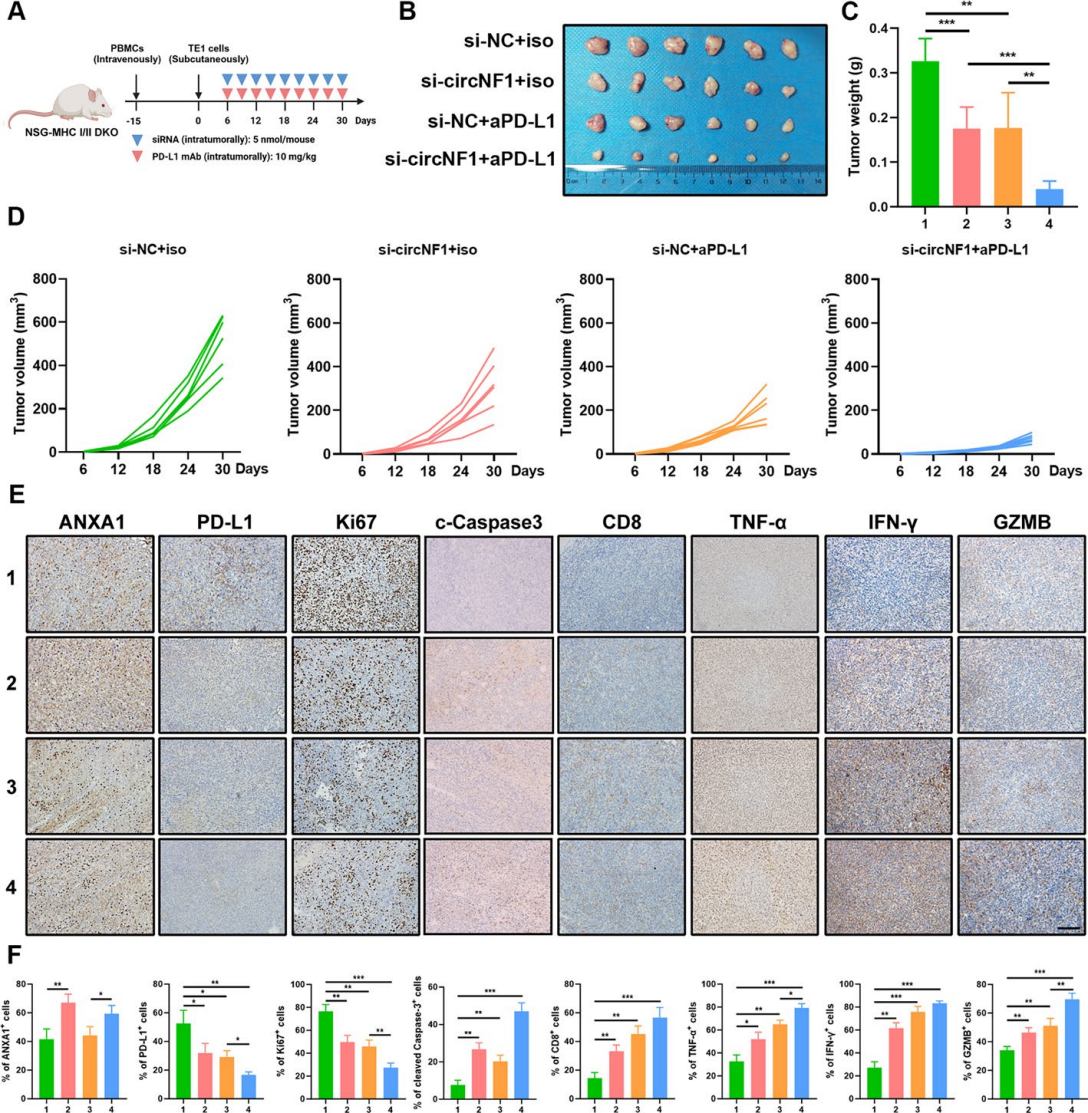
Combination of circNF1 blockade and anti-PD-L1 therapy exhibits favorable antitumor activity in vivo. **A** Schematic diagram of the experimental design of TE1 tumor-bearing humanized mouse model. *B* Macroscopic image of excised ESCC tumors from indicated groups. **C**, **D** The tumor weights (**C**) and growth curves (**D**) of tumor volume after initial implantation in each group. **E**, **F** Representative IHC images (**E**) and statistical quantitation (**F**) of immunostaining of ANXA1, PD-L1, Ki67, cleaved Caspase-3, CD8, TNF-α, IFN-γ, and GZMB in xenograft tumor tissues. Corresponding 1–4 groups are si-NC+iso, si-circNF1+iso, si-NC+αPD-L1, and si-circNF1+αPD-L1, respectively. Scale bar: 500 μm. The data are presented as mean ± SD. *P < 0.05, **P < 0.01, ***P < 0.001

We then attempted to explore the effect of circNF1 on PD-L1 checkpoint blockade therapy, with important implications for introducing circNF1 as a potential therapeutic target to improve immunotherapy efficacy. Owing to the lack of murine-derived ESCC cells, we intravenously injected human-derived PBMCs into NSG-MHC I/II DKO mice to mimic the immune microenvironment. A total of 15 days after implantation of human-derived PBMCs, we established subcutaneous xenografts of TE1 cells in NSG mice. When the tumor volume reached about 50 mm^3^, the NSG mice were divided into four groups and treated according to the corresponding strategy (Fig. 7A). We observed that intratumoral injection of genOFFTM in vivo siRNA targeting circNF1 remarkably retarded ESCC tumor growth. More importantly, the combination of siRNA targeting circNF1 with anti-PD-L1 showed the most pronounced decrease in tumor volume over time, suggesting that circNF1 deletion rendered ESCC cells more sensitive to anti-PD-L1 treatment (Fig. 7B–D). IHC staining of tumor tissues was performed, and the results demonstrated that the co-administration of siRNA targeting circNF1 and anti-PD-L1 noticeably declined Ki67 and elevated cleaved Caspase-3 level. It increased CD8^+^ T-cell infiltration and the secretion of cytotoxicity-associated molecules, including GZMB, IFN-γ, and TNF-α (Fig. 7E and F). In conclusion, these results suggested that circNF1 deletion reinforced the antitumor immunity of PD-L1 blockade.

### CircNF1 is correlated with the efficacy of PD-L1 mAb therapy in patients with ESCC

**Fig. 8:**
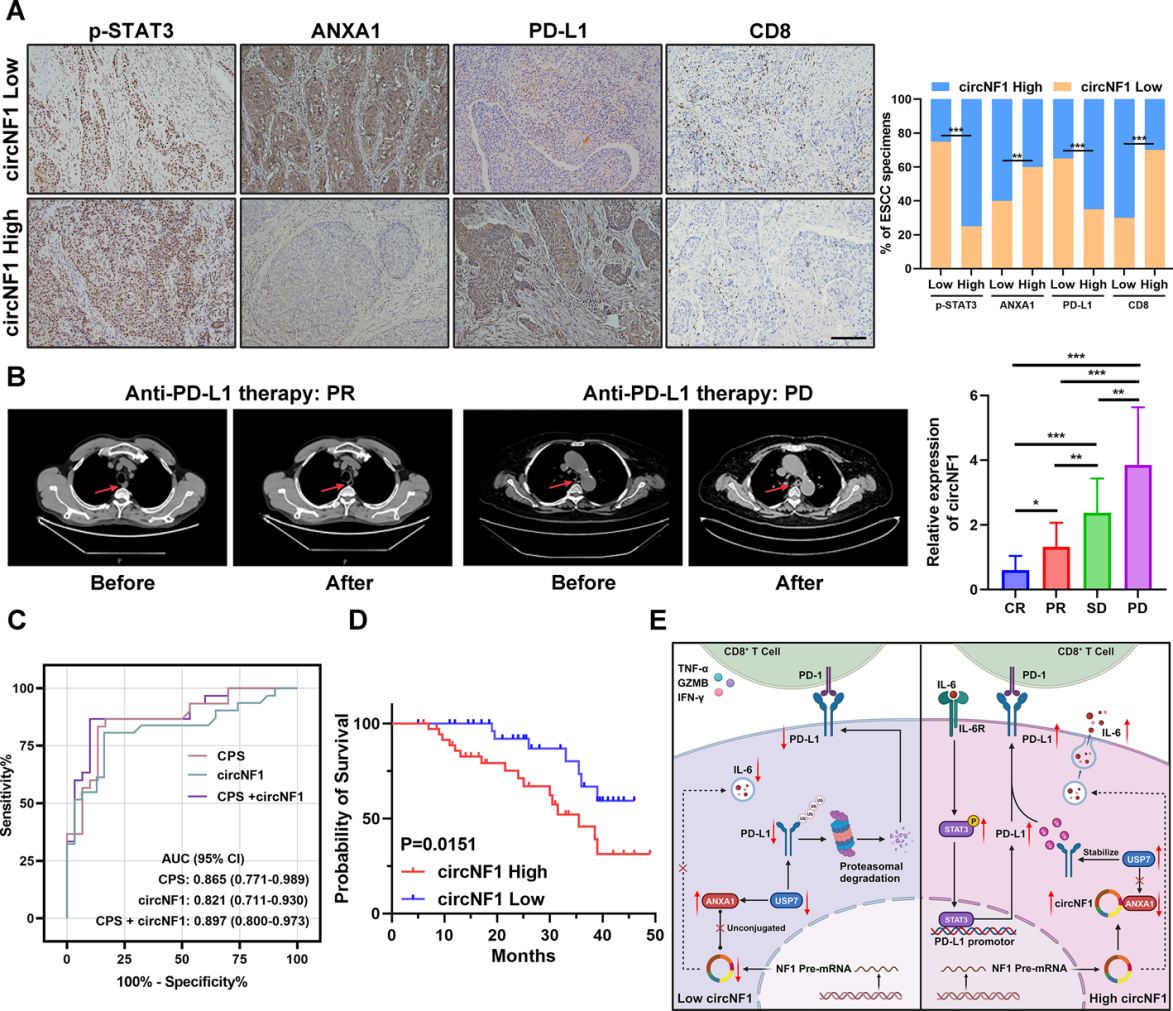
High expression of circNF1 negatively correlates with anti-PD-L1 therapy efficacy and prognosis of patients with ESCC. **A** Representative IHC images and statistical analysis of p-STAT3, ANXA1, PD-L1, and CD8 expression in ESCC tissues with different grades of circNF1. Scale bar: 100 μm. **B** Chest CT imaging of patients demonstrating PR or PD reaction after anti-PD-L1 treatment (the arrows showed the foci area). Quantification analysis of circNF1 expression in patients with ESCC with different treatment response in cohort 3. **C** ROC curves of the predicted circNF1 expression, CPS, and composite models combining circNF1 and CPS for predicting immunotherapy response. **D** Correlation analysis between circNF1 expression and overall survival of patients with ESCC. **E** Schematic summarizing the circNF1/ANXA1/USP7/PD-L1 axis and its role in modulating ESCC progression and immunosuppression. *P < 0.05, **P < 0.01, ***P < 0.001

To demonstrate the clinical relevance of the circNF1-involved signaling axis, we first assessed the protein expression levels of p-STAT3, ANXA1, PD-L1, and CD8 in 40 ESCC samples of cohort 1. Patients were classified into high and low groups on the basis of the circNF1 mRNA expression. Our IHC results revealed that a high level of circNF1 was positively correlated with lower ANXA1 expression and elevated p-STAT3 and PD-L1 expression, accompanied by declined CD8^+^ T cell infiltration in tumor microenvironment (Fig. 8A and Supplementary Table. S5). We further measured the serum level of circNF1 in cohort 3 containing 60 patients after anti-PD-L1 treatment, and observed the gradual decline in circNF1 along with improving response to immune checkpoint blockade. In particular, evaluating the computed tomography (CT) imaging and blood test results demonstrated that circNF1 expression in patients who achieved complete response (CR) and PR was substantially lower than that in patients with stable disease (SD) and PD (Fig. 8B). We analyzed the clinical value of circNF1 expression and combined positive score (CPS) of PD-L1 expression in prediciting response to anti-PD-L1 immunotherapy. The predictive accuracy of circNF1 and CPS was similar; the AUCs were calculated to be 0.821 and 0.865, respectively. When combining circNF1 level and CPS into an integrative model, the AUC achieved was 0.897, which noticeably improved the credibility for immunotherapy response prediction (Fig. 8C). Moreover, patients with high cricNF1 levels tended to have worse overall survival (Fig. 8D). Collectively, these clinical data supported our preclinical findings and indicated that circNF1 may serve as a novel predictive immunotherapy and diagnostic biomarker for ESCC (Fig. 8E).

## Discussion

Early symptoms of ESCC are often easily overlooked, with around 70% of esophageal cancer patients having locally advanced disease by the time they are diagnosed [15]. The advent of preoperative neoadjuvant therapy, including immune checkpoint inhibitors, as represented by PD-1/PD-L1 inhibitors, has revolutionized ESCC treatment, yet the overall prognosis remains poor. Therefore, uncovering effective biomarkers and exploring ways to enhance the efficacy of immune checkpoint inhibitors are critical for patients with esophageal cancer. In the present study, we identified for the first time that circNF1, which was highly expressed in ESCC tissues and serum, affected ESCC malignant progression and antitumor immunity. Mechanistically, we expanded in depth from the synthesis and degradation process of PD-L1, respectively, and determined that circNF1 upregulates PD-L1 levels by phosphorylating STAT3 to promote PD-L1 transcription as well as enhancing USP7-mediated deubiquitination of PD-L1.

Circular RNAs are a class of endogenous noncoding RNAs that are universal, conserved, and tissue-specific and have become a hot topic in the field of tumor biology. CircNF1 is induced by eukaryotic initiation factor 4A-III (EIF4A3) in breast cancer and acts as a competitive endogenous RNA to activate the Rat sarcoma (RAS) pathway and facilitate the malignant progression of breast cancer [16]. In addition, studies have indicated that circNF1 plays a tumor-promoting role in gastric cancer and glioblastoma [17, 18]. Interestingly, we found that circNF1 was tightly related to the therapeutic efficacy of anti-PD-L1 immunotherapy according to the microarray results, which prompted us to explore the biological function of circNF1 in ESCC. We obtained high circNF1 expression in ESCC tissues and serum and observed dramatic circNF1-triggered changes in ESCC cell proliferation and metastasis in ESCC cells. It is worthwhile to study in depth that subsequent RNA-seq identified the JAK–STAT3 pathway as the downstream regulatory pathways of circNF1. As one of the central communication nodes in cellular functions, the JAK–STAT3 signaling pathway is involved in various physiological functions such as cell proliferation, survival, and inflammation [19]. Aberrant JAK–STAT3 transduction activates proinflammatory cytokine signaling and disrupts immune homeostasis, leading to autoimmune diseases and cancer development [20]. STAT3 is an essential member of the STAT family, and abnormal STAT3 activity not only promotes tumor cell growth but also induces the expansion of tumor-supporting fibroblasts and inhibits antitumor immune responses [21]. In addition, phosphorylated STAT3 transfers to the nucleus and initiates transcription by binding to the promoter sequence of the target gene, thus fulfilling its function [22]. However, the regulatory relationship between circNF1 and STAT3 has not yet been reported. Here, using cytokine microarray, we verified that circNF1 caused ESCC cells to secrete more IL-6, a recognized cytokine for activating the JAK2/STAT3 axis, inferring that IL-6 acted as a key intermediate factor in circNF1-interacted JAK–STAT3 signaling pathway [23]. Subsequent studies revealed that the drive for ESCC progression by abnormally high circNF1 expression was achieved in part by increasing the phosphorylation level of STAT3. Although our study established an association between circNF1 and the JAK–STAT3 signaling pathway in ESCC, the approach by which circNF1 promotes IL-6 in ESCC cells remains unclear. Previous studies have suggested that some circRNAs competitively bind miRNAs to increase IL-6 secretion by stabilizing IL-6 mRNA. For example, in gastric cancer cells, circRBM33 targets miR-149 to manipulate IL-6 expression [24]. In addition, Zhou et al. reported that the circular RNA cSERPINE2 could promote IL-6 secretion by activating the NF-κB pathway, implying the existence of other circRNA-mediated pathways to regulate IL-6 secretion [25]. However, current reports on this issue are limited, thereby necessitating further research.

To date, the biological roles of circRNAs have been extensively investigated, the most classical of which is acting as microRNA sponges or decoys. Nevertheless, the functional diversity of circRNAs still needs to be comprehensively explored. CircRNAs have been reported to bind to proteins relying on their own conserved protein-binding sequences, thereby regulating protein subcellular localization, stability, and the formation of multicomplexes [26]. For example, circNEIL3 blocks the binding of IGF2BP3 to the E3 ubiquitin ligase HECTD4 via steric hindrance, ultimately inhibiting the ubiquitination of IGF2BP3 [27]. Notably, we found a similar role in circNF1 in ESCC, which competes with USP7 for the same binding domain of ANXA1. USP7, also known as HAUSP, is one of the deubiquitinating enzymes in the ubiquitin–proteasome system. USP7 removes ubiquitin molecules from substrates, preventing substrate degradation and resulting in their stabilization, which in turn modulates a wide range of cellular processes, such as the DNA damage response, immune response, cellular transcription, epigenetic inheritance, and viral infection [28]. Owing to co-competition for the TRAF binding domain in ANXA1, circNF1 impedes the interaction of USP7 with ANXA1, leading to increased levels of ubiquitination of ANXA1 and reduced protein stability. ANXA1 expression in tumors was found to be tissue-specific, as evidenced by increased levels in lung, liver, and colorectal cancers, while it showed decreased levels in prostate, nasopharyngeal, and oral squamous cell carcinomas [29–34]. However, there are conflicting studies on the expression and biological role of ANXA1 in ESCC. Wang et al. revealed that high expression of ANXA1 is prevalent in esophageal and esophagogastric junction adenocarcinomas, which is associated with more advanced pathologic T stage and the presence of distant metastases and is an independent prognostic factor for patient survival [35]. Furthermore, Han et al. found that ectopic expression of ANXA1 promoted ESCC proliferation and invasion [36]. Such results confirm the role of ANXA1 as an oncogene in ESCC. A different view was later presented by Chen et al., who used normal epithelium, precancerous lesions, and cancerous tissues originating from the same individual esophagus and detected a progressive decline in ANXA1 expression as the ESCC evolved [37]. Our findings were more supportive of the latter idea, according to which the oncogenic factor circNF1 spatially blocked the binding of ANXA1 and USP7, increased the ubiquitination of ANXA1, and promoted its degradation through the ubiquitin–proteasome system. Functionally, decreased ANXA1 compensated for ESCC malignant proliferation, migration, and invasion induced by circNF1 knockdown, confirming that ESCC cells drive cancer development and metastasis by upregulating circNF1 to impair ANXA1 expression. Herein, we revealed the molecular mechanism whereby ANXA1 protein stability was dynamically regulated, as well as the new evidence for circRNA-mediated protein ubiquitination in ESCC.

Highly expressed PD-L1 binds to its receptor PD-1 and thus prevents T cell activation and induces T cell apoptosis, which is a way for tumor cells to achieve immune escape [38]. It is reported that the PD-L1 positivity rate in esophageal adenocarcinoma is only 18%, whereas the PD-L1 positivity rate in ESCC is approximately 44%, suggesting that ESCC may be more responsive to immunotherapy [39]. Therefore, an in-depth investigation of how PD-L1 accumulates in ESCC will help control the progression of ESCC and improve the efficacy of immunotherapy in patients with ESCC. Recent reports have elucidated the different mechanisms by which PD-L1 abundance is regulated in tumors, which can be broadly grouped into the following four points: genomic alterations, epigenetic regulation, transcriptional regulation, and posttranslational modifications [40]. However, the regulatory mechanisms of PD-L1 expression in ESCC have not been fully elucidated with respect to the currently available studies. Although STAT3 activation has been shown to stimulate PD-L1 expression in ESCC, their direct interaction has not been clearly articulated. Here, we first found by ChIP experiments that the circNF1-activated JAK–STAT3 signaling pathway promoted p-STAT3 binding to the promoter of PD-L1, which accelerated PD-L1 transcription. This provided evidence that circNF1 upregulated PD-L1 expression at the transcriptional level. Next, under the condition that the presence of circNF1 prevented USP7 from binding to ANXA1, we investigated the effect of USP7 on PD-L1 ubiquitination. Strikingly, the weakened binding of USP7 to ANXA1 instead induced enhanced binding of USP7 to PD-L1. This event inevitably led to reduced degradation of PD-L1 via the ubiquitin–proteasome system, which proved that circNF1 upregulated PD-L1 expression through ubiquitination modification. PD-L1 inhibitors, mainly durvalumab, atezolizumab, and bavencio, are rapidly becoming a therapeutic option for cancers that exhibit PD-L1 overexpression during treatment [41]. However, the efficacy of single-drug therapy is insufficient to meet clinical needs, and there is a need to combine existing therapeutic approaches and actively explore new targets and combinations. This prompted us to observe the effect of circNF1 on antitumor immunity in ESCC cells. Our in vitro experiments showed reduced growth, increased apoptosis of PBMCs, and diminished killing ability of CD8^+^ T cells after co-culture with ESCC cells overexpressing circNF1. Moreover, the deletion of circNF1 by siRNA not only increased CD8^+^ T cell-mediated cytotoxicity against tumor cells but also rendered ESCC cells more sensitive to PD-L1 checkpoint blockade therapy in tumor-bearing NSG mice models. This suggests that PD-L1 upregulation in ESCC cells results in blocked activation and reduced infiltration of CD8^+^ T cells, which may be one of the reasons for the excessive growth of ESCC cells. It is worth noting that we used only a single cell line to establish the animal model in vivo. Further validation with other ESCC cell lines would be more convincing. In addition, since other immune cells within PBMCs, such as monocytes, B cells, and natural killer (NK) cells, also directly or indirectly damage tumor cells through secreting cytokines, isolating T cells for future investigations would provide more precise insights into their specific contributions.

After recent years of research, circRNA molecules with specific changes have been identified in a variety of tumors that serve as potential therapeutic targets [42]. Currently, several nucleic acid drugs for manipulating circRNAs have been investigated in the clinical setting, such as siRNAs and antisense oligonucleotides (ASOs) [43]. The key point is how to deliver these nucleic acid drugs precisely to the target site under the premise of maintaining sequence stability. Lipid nanoparticles (LNPs) are among the earliest applied delivery systems composed of phospholipid bilayers. By encapsulating mini-nucleic acid drugs in LNPs, it is possible to protect these drugs from degradation and clearance, and facilitate their transport across the cell membrane to the target site [44]. In addition, nucleic acid self-assembled nanocarriers can efficiently load nucleic acid aptamers through base complementary pairing and carry targeted proteins to improve the selectivity of drug delivery. Therefore, targeting circNF1 to augment the efficacy of PD-L1 inhibitors for immunotherapy in patients with ESCC is potentially achievable in the future.

## Conclusions

Overall, our work validated for the first time the function of circNF1 as a pro-oncogenic circRNA in ESCC and established a critical association between circNF1 and PD-L1-mediated immune escape. CircNF1 controlled PD-L1 levels not only by increasing STAT3-induced PD-L1 transcription but also by inhibiting the ANXA1- and USP7-mediated ubiquitination of the PD-L1 protein. We elucidated the role of circNF1 in PD-L1-mediated immune escape to enhance tumorigenicity and identified circNF1 as a predictive marker of the efficacy of PD-L1 immune checkpoint therapy in patients with ESCC. In conclusion, the current study provides a theoretical basis for selecting concomitant therapies for anti-PD-L1 in the ESCC clinical setting.

## Supplementary Information

Supplementary file 1.

## Data Availability

All data generated or analyzed during this study are included in this published article and the supplemental information. Data generated from RNA sequencing are deposited in the Figshare (https://figshare.com/s/d5c7292621bfb4fa3d5d).

## Abbreviations

circRNAs: Circular RNA
ESCC: Esophageal squamous cell carcinoma
siRNA: Small interfering RNA
CCK-8: Cell counting kit-8
TUNEL: TdT-mediated dUTP Nick-End labeling staining
ROC: Receiver operating characteristic curves
AUC: Area under curve
TNF-α: Tumor necrosis factor alpha
IFN-γ: Interferon gamma
GZMB: Granzyme B
