# Supplementary material for: CircNF1 modulates the progression and immune evasion of esophageal squamous cell carcinoma through dual regulation of PD-L1
Source: Cell Mol Biol Lett. 2025 Mar 29;30:37. doi: 10.1186/s11658-025-00712-y (PMC11955112; doi:10.1186/s11658-025-00712-y)
Supplement: Supplementary file 1 [file 11658_2025_712_MOESM1_ESM.docx]

**Supplementary information**

**CircNF1 modulates the progression and immune evasion of esophageal squamous cell carcinoma through dual-regulation of PD-L1**

Chang Wang, Chenxi Ju, Dan Du, Peiyu Zhu, Jie Yin, Jinlin Jia, Xue Wang, Xinyu Xu, Li Zhao, Junhu Wan, Ting Sun, Lijun Yang, Hongle Li, Fucheng He, Mingxia Zhou, Jing He

**Table of contents:**

**Supplementary Figures 1-7**

**Supplementary Tables 1-5**

**
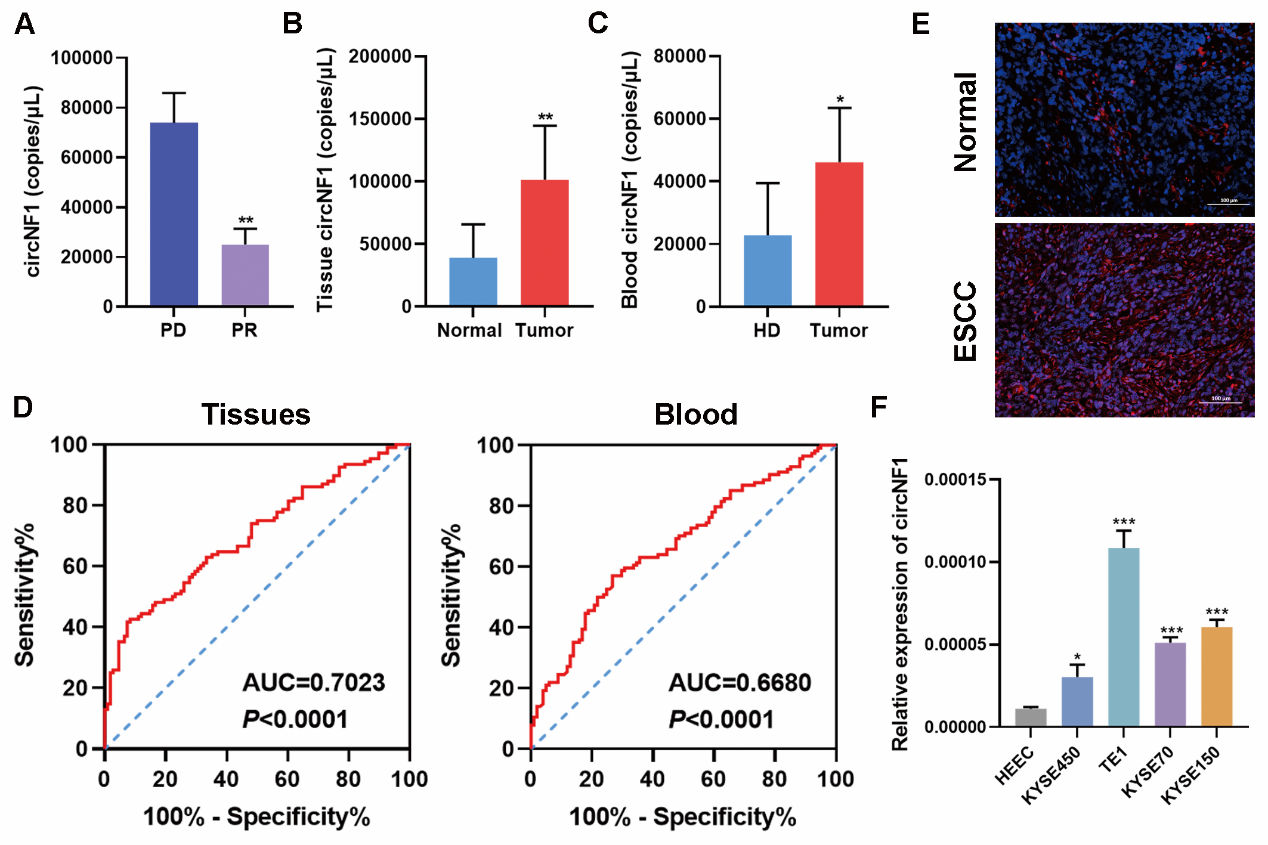
**

**Fig. S1: CircNF1 is highly expressed in ESCC.**

(A) The circNF1 level was quantified in ESCC patients showing PD or PR after anti-PD-L1 treatment by ddPCR. (B) Absolute quantification of circNF1 in ESCC tissues (n=8) and paired esophagus tissues (n=8) by ddPCR. (C) Absolute quantification of circNF1 in the serum of ESCC patients (n=8) and healthy donors (n=8) by ddPCR. (D) The ROC curve analyzing the diagnostic value of circNF1 in ESCC tissue (left panel) and serum (right panel). (E) FISH images showing circNF1 abundance in ESCC and paracancerous tissues. Scale bar: 100 μm. (F) qRT-PCR detecting circNF1 levels in ESCC cell lines. *P < 0.05, **P < 0.01.

**
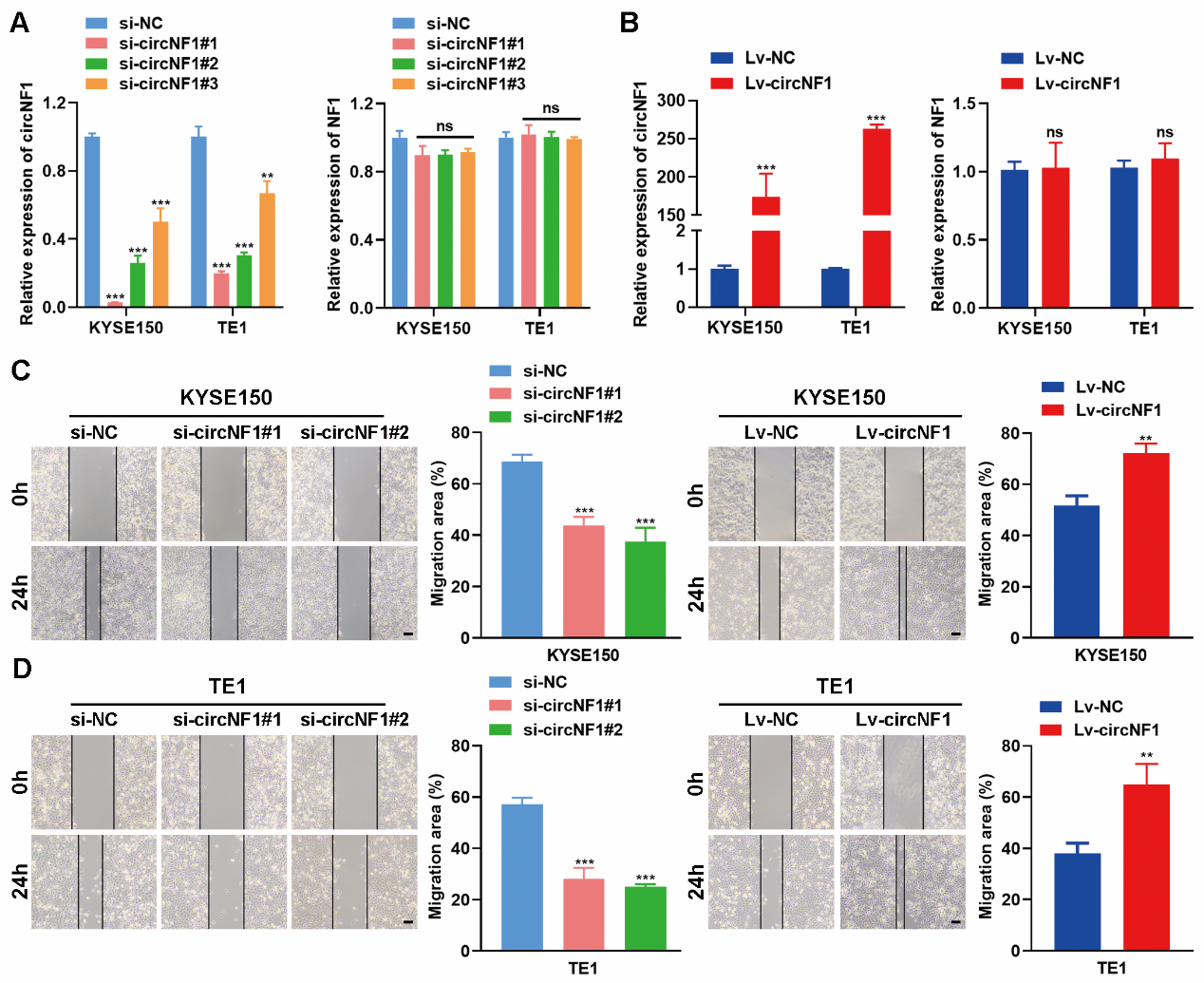
**

**Fig. S2: Validation of circNF1 knockdown and overexpression efficiency and detection of ESCC cell migration ability.**

(A, B) qRT-PCR analysis showing the expression of circNF1 (left panel) and NF1 mRNA (right panel) in ESCC cells transfected with (A) si-NC or si-circNF1, and infected with (B) Lv-NC or Lv-circNF1. (C, D) Wound healing assays examining the migration abilities of ESCC cells after circNF1 knockdown and overexpression. Scale bar: 200 μm. **P < 0.01, ***P < 0.001. ns, not significant.

**
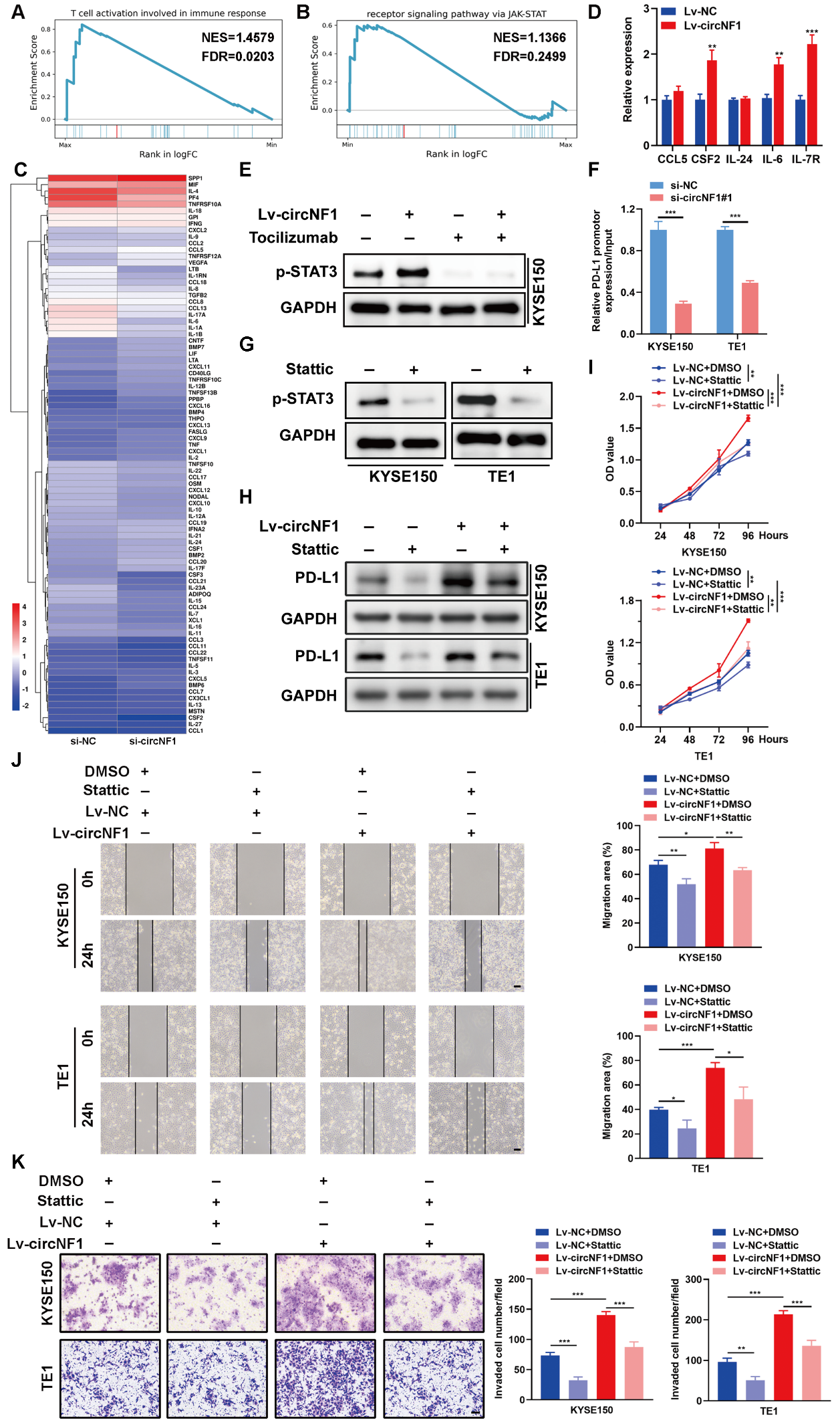
**

**Fig. S3: Stattic attenuates circNF1-induced ESCC malignant behaviors and PD-L1 level.**

(A, B) GSEA enrichment plots showing the enrichment of T cell activation involved in immune response (A) and the JAK-STAT signaling pathway (B) in circNF1-regulated targets. (C) PCR array showing alterations in mRNA levels of multiple cytokines. (D) qRT-PCR validating the expression of five candidate genes involved in the JAK-STAT3 pathway in circNF1-overexpressed ESCC cells. (E) Western blotting analysis of p-STAT3 protein level after overexpressing circNF1 or Tocilizumab treatment. (F) qRT-PCR validating the binding of p-STAT3 and PD-L1 promoter. (G) Western blotting analysis of p-STAT3 protein level after Stattic treatment. (H) Western blotting analysis of PD-L1 protein level after overexpressing circNF1 or Stattic treatment in ESCC cells. (I) CCK-8 assays showing the proliferation ability of ESCC cells after overexpressing circNF1 or Stattic treatment. (J, K) Detection of the metastatic ability of ESCC cells after overexpressing circNF1 or Stattic treatment by wound-healing (J) and Transwell invasion (K) assays. Scale bar: 200 μm (G) or 100 μm (H). The data are presented as mean ± SD. *P < 0.05, **P < 0.01, ***P < 0.001.

**
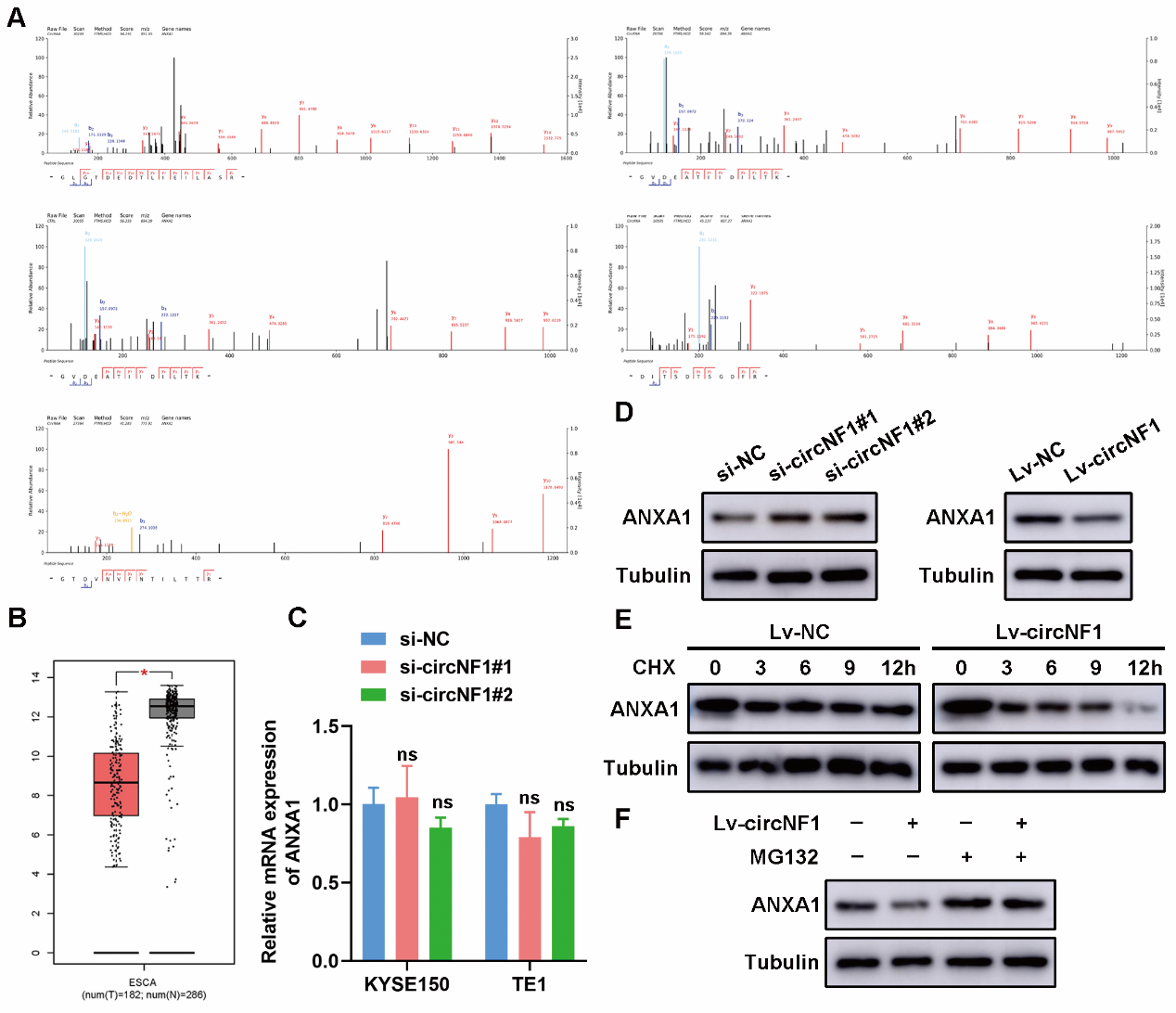
**

**Fig. S4: CircNF1 inhibits ANXA1 expression by promoting its protein degradation.**

(A) Secondary mass spectrometry of the remaining five peptides of ANXA1. (B) The expression profile of ANXA1 in ESCA (Esophageal carcinoma) was extracted from GEPIA database. (C) Detection of mRNA level of ANXA1 in ESCC cells with circNF1 knockdown. (D) Western blotting images of ANXA1 protein in TE1 cells knocking down or overexpressing circNF1. (E) Western blotting showing the effect of overexpressing circNF1 on ANXA1 protein stability in TE1 cells treated with 20 µg/mL CHX for indicated times. (F) Western blotting analyzing ANXA1 protein level after treatment with proteasome inhibitor MG132 in circNF1-overexpressed TE1 cells. The data are presented as mean ± SD. ns, not significant.

**
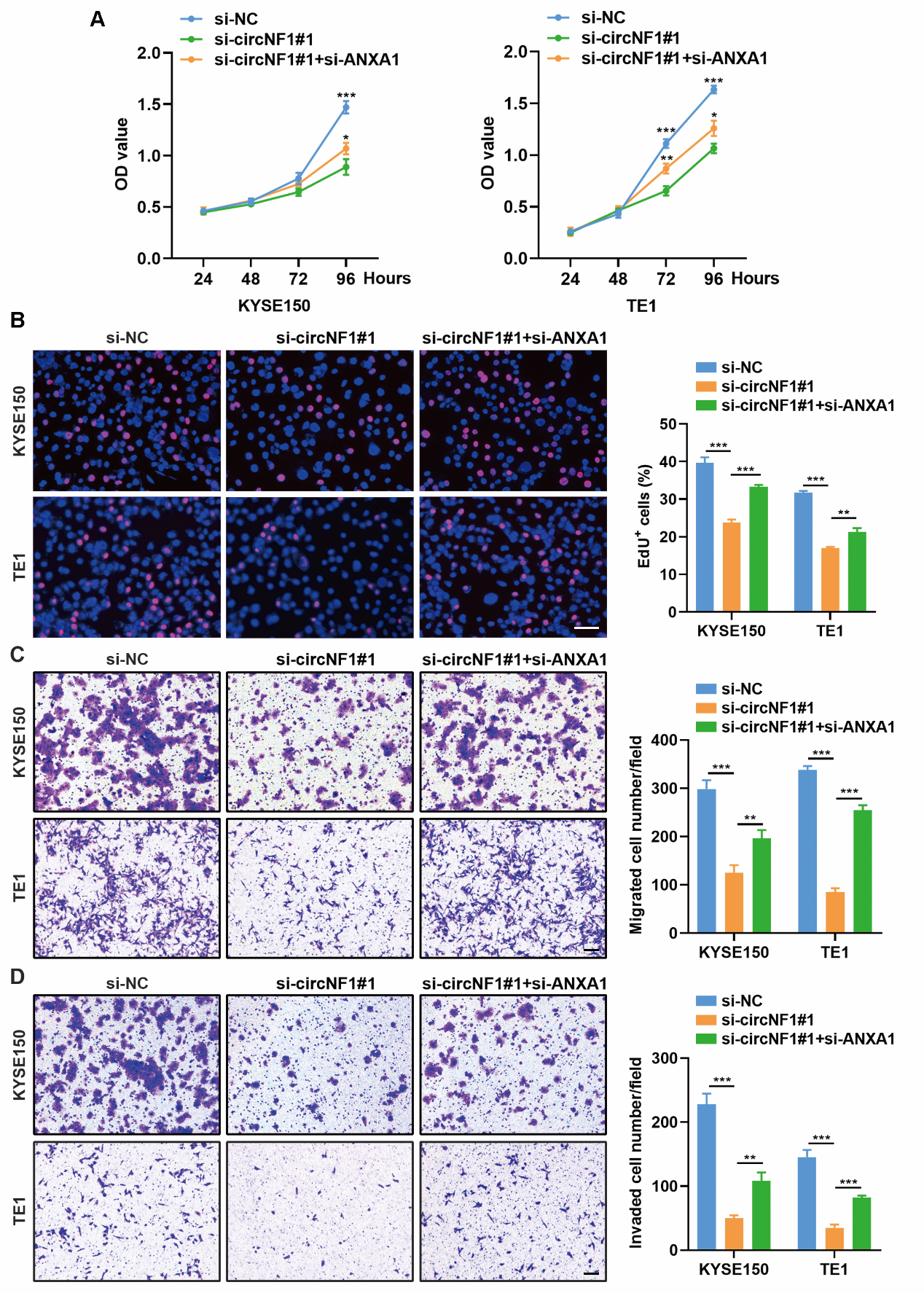
**

**Fig. S5: CircNF1 modulates the malignant biological behavior of ESCC by targeting ANXA1.**

(A, B) CCK-8 (A) and EdU assays (B) observing cell proliferation after transfecting si-circNF1 with/without si-ANXA1 in ESCC cells. Scale bar: 100 μm. (C, D) Transwell migration (C) and invasion (D) assays for ESCC cells transfected si-circNF1 with/without si-ANXA1. Scale bar: 100 μm. The data are presented as mean ± SD. *P < 0.05, **P < 0.01, ***P < 0.001.

**
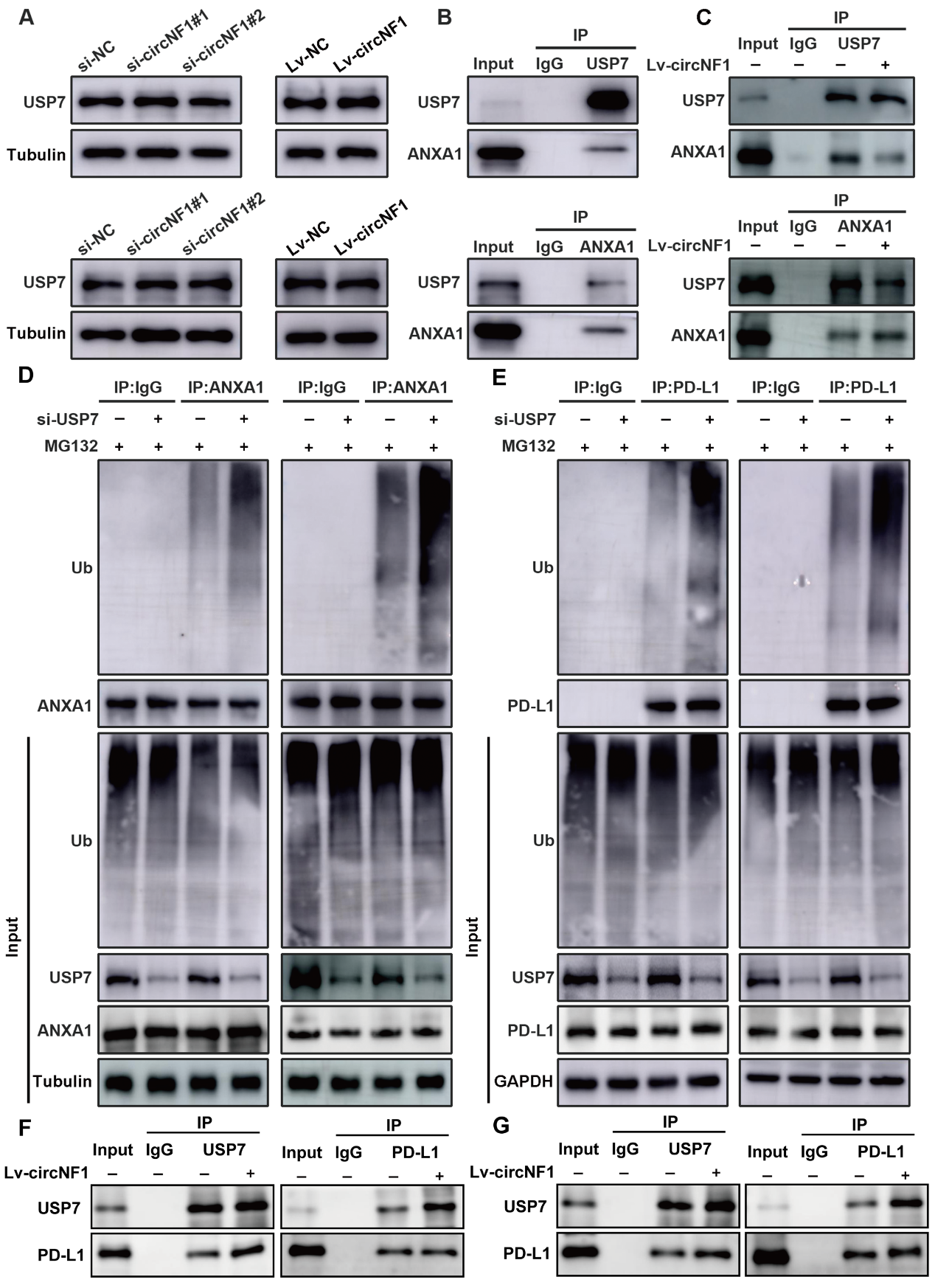
**

**Fig. S6: USP7 serves as a deubiquitinating enzyme for both ANXA1 and PD-L1.**

(A) Western blotting detecting the effect of circNF1 knockdown and overexpression on USP7 protein level in KYSE150 (upper panel) and TE1 (bottom panel) cells. (B) Co-IP analysis of endogenous interaction between ANXA1 and USP7 with USP7 (upper panel) or ANXA1 (bottom panel) antibodies in TE1 cells. (C) Co-IP assays revealing the interaction between ANXA1 and USP7 in circNF1-overexpressed TE1 cells. (D, E) Co-IP assays verifying the effects of USP7 knockdown on ANXA1 (D) and PD-L1 (E) polyubiquitination level in KYSE150 (left panel) and TE1 (right panel). (F, G) Co-IP assays revealing the interaction between USP7 and PD-L1 in circNF1-overexpressed KYSE150 (left panel) and TE1 (right panel).

**
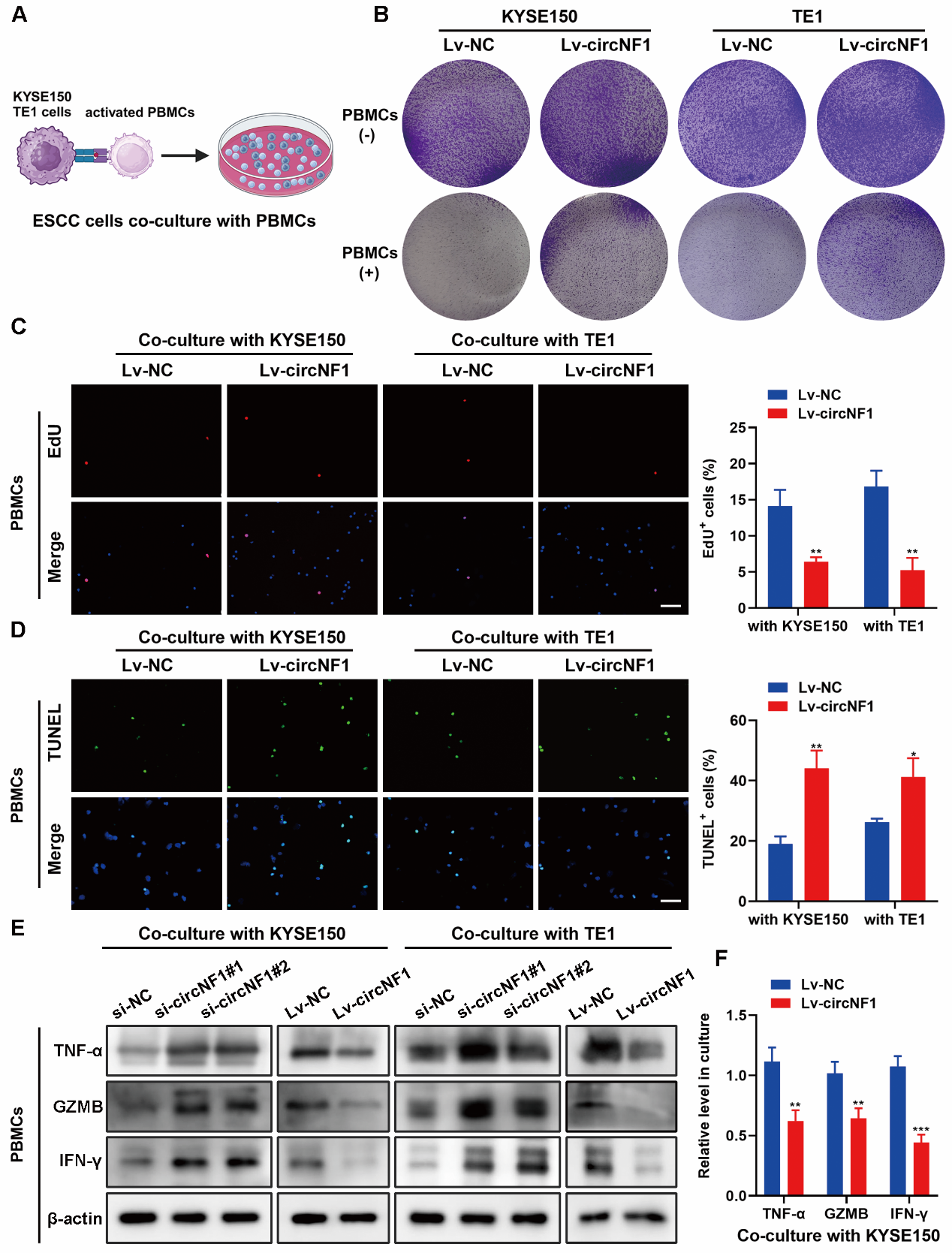
**

**Fig. S7: CircNF1 suppresses T cell cytotoxicity against ESCC cells.**

(A) The schematic representation of ESCC cells co-cultured with PBMCs. (B) Activated CD8^+^ T cell co-cultured with Lv-NC or Lv-circNF1 ESCC cells to assess the T cell killing activity. (C) EdU assays investigating the proliferation of PBMCs after co-culturing with circNF1-overexpressed ESCC cells. Scale bar: 100 μm. (D) TUNEL assays detecting the apoptosis of PBMCs after co-culturing with circNF1-overexpressed ESCC cells. Scale bar: 100 μm. (E) Western blotting analyzing the protein levels of TNF-α, GZMB, and IFN-γ secreted by activated T cells co-cultured with circNF1 knockdown or overexpressing ESCC cells. (F) ELISA assays detecting the abundance of TNF-α, GZMB, and IFN-γ in culture supernatants of T cells co-cultured with overexpressing KYSE150 cells. The data are presented as mean ± SD. *P < 0.05, **P < 0.01, ***P < 0.001.

**Table S1. The siRNA sequence for knocking down the indicated RNAs or proteins.**

| Gene name | Sequences |
| --- | --- |
| si-circNF1#1 | CAACATGAATAAGCTTCCA |
| si-circNF1#2 | ACATGAATAAGCTTCCAAT |
| si-circNF1#3 | CATGAATAAGCTTCCAATA |
| si-USP7 | GAATGACATGTACGATGAA |
| si-ANXA1 | GCGTCAACAGATCAAAGCA |

**Table S2. Forward and reverse primers for qRT-PCR.**

|  | Forward | Reverse |
| --- | --- | --- |
| circNF1-D | ATGAAAACAACATGAATAAGC | TTTTCAGCAGCTTCTCCAAATA |
| circNF1-C | GCTGGTCAAACAGTTGCTGC | TTGAAGTTGTTGCAGCTGAGAG |
| NF1 | CGAATCATCACCAATTCCGCA | CCACAACCTTGCACTGCTTTAT |
| CCL5 | GCTGCTTTGCCTACATTGCC | TCGGGTGACAAAGACGACTG |
| CSF2 | CCGGAAACTTCCTGTGCAAC | GTCTCACTCCTGGACTGGCT |
| IL-24 | GCCTTCTGGGCTGTGAAAGA | CAGGGTGTGGACAAGGTAACA |
| IL-6 | TTCGGTCCAGTTGCCTTCTC | CTGAGATGCCGTCGAGGATG |
| IL-7R | CCAACCGGCAGCAATGTATG | AGGATCCATCTCCCCTGAGC |
| PD-L1 promoter | CAAGGTGCGTTCAGATGTTG | GGCGTTGGACTTTCCTGA |
| ANXA1 | GCGGTGAGCCCCTATCCTA | TGATGGTTGCTTCATCCACAC |
| GAPDH | CAGGAGGCATTGCTGATGAT | GAAGGCTGGGGCTCATTT |

D: divergent primer C: convergent primer

**Table S3. Antibodies used in this study.**

| Antibody | Source | Identifier |
| --- | --- | --- |
| Phospho-Stat3 (Tyr705) mAb | Cell Signaling Technology | #9145 |
| Stat3 Rabbit mAb | HUABIO | ET1607-38 |
| IgG Rabbit mAb | Abcam | ab172730 |
| Anti-CD8 alpha antibody | Abcam | ab217344 |
| Cleaved Caspase-3 Antibody | Cell Signaling Technology | #9661 |
| PD-L1 Monoclonal antibody | Proteintech | 66248-1-Ig |
| Annexin A1 Polyclonal antibody | Proteintech | 21990-1-AP |
| ubiquitin Polyclonal antibody | Proteintech | 10201-2-AP |
| USP7 Monoclonal antibody | Proteintech | 66514-1-Ig |
| TNF Alpha Polyclonal antibody | Proteintech | 17590-1-AP |
| Granzyme B antibody | BIOSS | bs-1351R |
| IFN Gamma Polyclonal antibody  Anti-Ki67 antibody | Proteintech  Abcam | 15365-1-AP  ab15580 |
| GAPDH Polyclonal antibody | Proteintech | 10494-1-AP |
| Alpha Tubulin Polyclonal antibody | Proteintech | 11224-1-AP |
| DYKDDDDK tag antibody | Proteintech | 80010-1-RR |
| MYC tag Polyclonal antibody | Proteintech | 16286-1-AP |

**Table S4. The relationship between circNF1 expression and clinicopathological variables in ESCC patients.**

|  | circNF1 expression | | P-value |
| --- | --- | --- | --- |
|  | High (n=54) | Low (n=54) |  |
| **Gender** |  |  |  |
| Male | 33 | 28 | 0.3318 |
| Female | 21 | 26 |  |
| **Age** |  |  |  |
| ≤ 65 years | 24 | 26 | 0.6995 |
| > 65 years | 30 | 28 |  |
| **Tumor size** |  |  |  |
| ≤ 3 cm | 24 | 33 | 0.0828 |
| > 3 cm | 30 | 21 |  |
| **Differentiation grade** |  |  |  |
| G1+G2 | 27 | 30 | 0.5631 |
| G3 | 27 | 24 |  |
| **Depth of Tumor invasion**  T1  T2+T3+T4  **Lymphatic metastasis** | 21  33 | 22  32 | 0.8442 |
| N0+N1 | 19 | 32 | **0.0122*** |
| N2+N3 | 35 | 22 |  |
| **TNM stage** |  |  |  |
| I+II | 22 | 31 | 0.0832 |
| III+IV | 32 | 23 |  |

**Table S5.** **The mRNA level of circNF1 and the protein level of p-STAT3, PD-L1 and CD8 in ESCC patients.**

| Patient ID | CircNF1 | P-STAT3 | PD-L1 | CD8 |
| --- | --- | --- | --- | --- |
| 2 | Low | Low | High | Low |
| 3 | Low | Low | High | Low |
| 5 | High | High | Low | High |
| 9 | Low | Low | Low | High |
| 10 | High | Low | Low | Low |
| 14 | Low | Low | High | Low |
| 18 | Low | Low | Low | High |
| 22 | High | High | High | High |
| 23 | Low | Low | Low | Low |
| 24 | High | Low | Low | Low |
| 27 | High | High | Low | High |
| 34 | Low | Low | High | Low |
| 39 | High | High | Low | High |
| 41 | Low | High | Low | High |
| 44 | High | High | High | High |
| 45 | Low | Low | High | Low |
| 49 | High | Low | Low | Low |
| 50 | High | High | High | High |
| 53 | Low | Low | High | Low |
| 59 | High | Low | Low | Low |
| 62 | Low | High | Low | High |
| 64 | High | High | Low | Low |
| 65 | Low | Low | High | Low |
| 67 | Low | High | Low | High |
| 71 | High | High | Low | High |
| 78 | Low | Low | High | Low |
| 79 | High | Low | Low | Low |
| 82 | Low | High | Low | High |
| 85 | High | High | High | High |
| 86 | Low | Low | High | Low |
| 89 | High | High | Low | High |
| 90 | High | High | High | High |
| 91 | Low | Low | High | Low |
| 94 | Low | High | Low | High |
| 95 | High | High | High | High |
| 98 | Low | Low | High | Low |
| 100 | Low | Low | High | Low |
| 101 | High | High | High | High |
| 106 | High | High | Low | Low |
| 108 | High | High | High | High |
